# A mechanism underlying position-specific regulation of alternative splicing

**DOI:** 10.1093/nar/gkx901

**Published:** 2017-10-09

**Authors:** Fursham M. Hamid, Eugene V. Makeyev

**Affiliations:** 1 Centre for Developmental Neurobiology, King's College London, London SE1 1UL, UK; 2 School of Biological Sciences, Nanyang Technological University, Singapore 637551, Singapore

## Abstract

Many RNA-binding proteins including a master regulator of splicing in developing brain and muscle, polypyrimidine tract-binding protein 1 (PTBP1), can either activate or repress alternative exons depending on the pre-mRNA recruitment position. When bound upstream or within regulated exons PTBP1 tends to promote their skipping, whereas binding to downstream sites often stimulates inclusion. How this switch is orchestrated at the molecular level is poorly understood. Using bioinformatics and biochemical approaches we show that interaction of PTBP1 with downstream intronic sequences can activate natural cassette exons by promoting productive docking of the spliceosomal U1 snRNP to a suboptimal 5′ splice site. Strikingly, introducing upstream PTBP1 sites to this circuitry leads to a potent splicing repression accompanied by the assembly of an exonic ribonucleoprotein complex with a tightly bound U1 but not U2 snRNP. Our data suggest a molecular mechanism underlying the transition between a better-known repressive function of PTBP1 and its role as a bona fide splicing activator. More generally, we argue that the functional outcome of individual RNA contacts made by an RNA-binding protein is subject to extensive context-specific modulation.

## INTRODUCTION

Alternative pre-mRNA splicing expands protein diversity and gene regulation possibilities in eukaryotes through non-uniform utilization of exons and introns (1–4). It is extensively controlled by interactions between RNA-encoded *cis*-elements and cognate RNA-binding proteins (RBPs) (5–9).

Many RBPs can either activate or repress splicing depending on their binding position relative to a regulated exon (10–12). For example, transcriptome-wide crosslinking-immunoprecipitation (CLIP) studies combined with systematic analyses of splicing patterns showed that RBPs from the NOVA family often repress alternative exons when recruited to an upstream intronic position or the exon itself and promote their inclusion into mature mRNA when bound downstream (13,14). Correlation of upstream binding with repression and downstream binding with activation of alternative exons has been shown for several other RBPs including those from the Rbfox, Mbnl, Celf and Esrp families and possibly hnRNP L (15–22). Similar effects have been observed in minigene experiments relying on a recombinant tethering approach (23).

Two distinct possibilities may account for these molecular trends. An RBP can function invariantly, as a constitutive repressor or activator, but have opposite effects on different pre-mRNAs by targeting either regulated exons themselves or their splicing competitors. This mechanism has in fact been proposed to underlie at least a subset of position-specific splicing events (24,25). Alternatively, an RBP may repress some alternative exons and activate others in a direct manner, suggesting a possibility of a genuine switch between the two regulation modes. Possible contribution of this mechanism to splicing regulation has not been investigated systematically.

Polypyrimidine tract-binding protein 1 (PTBP1/PTB/hnRNP I) is an important example of a position-specific regulator of alternative splicing that targets a large number of transcripts in developing brain and muscle (12,26,27). Similar to the examples above, PTBP1 binding immediately upstream of or within a regulated exon correlates with skipping whereas recruitment to a downstream position tends to promote inclusion (25,28,29). Mechanistically, PTBP1 binding upstream of or within an exon can inhibit productive association of the core splicing factors U2 snRNP and U2AF with the branch point and the pyrimidine tract, respectively (30,31). At least in some cases, this may involve PTBP1-mediated repression of exon definition interactions (32).

How downstream recruitment stimulates inclusion of the regulated exon is understood to a substantially lesser extent. When bound to sufficiently distant intronic positions, PTBP1 may inhibit a downstream constitutive exon thus giving the regulated exon a competitive advantage (12,25). Conversely, PTBP1 recruited immediately downstream of a cassette exon or an alternative 5′ splice site (5’ss) has been proposed to activate them directly (28,33). However, the molecular mechanism underlying this hypothetical activity is unknown.

Our understanding of the PTBP1-dependent ‘splicing code’ is further complicated by recurring examples where PTBP1 promotes skipping of alternative exons by forming contacts with both upstream and downstream intronic sequences (34,35). Several models have been proposed to explain this effect including looping, lateral spreading of PTBP1 oligomers, recruitment of co-repressors or/and formation of unproductive splicing complexes (34–40). How these molecular events might relate to PTBP1-mediated splicing activation remains an open question.

Here we combine bioinformatics and experimental approaches to uncover a mechanism that allows PTBP1 to activate alternative exons in a direct manner. Our data also explain how recruitment of this protein both upstream and downstream of a regulated exon can cancel out the activation effect and lead to repression.

## MATERIALS AND METHODS

### Plasmids

AdML-M3 plasmid encoding an adenovirus-derived splicing substrate was a gift from Robin Reed (Addgene; #11244). U1-encoding expression vector pN/S6U1 was described earlier (41). New constructs were generated using standard molecular cloning techniques and enzymes from NEB, as described in Supplementary Table S3. Site-specific mutagenesis was done using KAPA HiFi DNA polymerase (KAPA Biosystems) and corresponding mutagenic primers (Supplementary Table S4). The plasmid maps and sequences are available on request.

### Cell cultures

CAD mouse neuroblastoma cells (42) were cultured in Dulbecco's Modified Eagle Medium/High Glucose (DMEM; Thermo Fisher Scientific/GIBCO), supplemented with 11% FetalClone III Serum (GE Healthcare/HyClone), 1 mM sodium pyruvate (Thermo Fisher Scientific/GIBCO), 100 IU/ml penicillin and 100 μg/ml streptomycin, at 37°C in the presence of 5% CO_2_. For transfection experiments, cells were plated in the CAD medium without antibiotics at a density of 4 × 10^5^ cells per well of a six-well plate for tissue culture. Twelve hours post-plating, cells were transfected with corresponding ON-TARGETplus siRNA (GE Healthcare/Dharmacon; siControl [D-001810-01-20]; siPtbp1 [J-042865-11-0050]; siPtbp2 [L-049626-01-0005]) using Lipofectamine RNAiMAX (Thermo Fisher Scientific/Invitrogen). Following 36-hour incubation, cell cultures were typically re-transfected with 1 μg of a minigene plasmid using Lipofectamine 2000 and incubated for another 36 h prior to RNA harvest. For U1 snRNA suppressor experiments, the ratio of pN/S6U1 plasmid to minigene plasmid used for transfections is 9:1. Neurons, neuronal progenitor cells and astrocytes were isolated from mouse cortices and maintained as described elsewhere (43).

### RT-PCR and RT-qPCR

Total RNA was harvested from adherent cells using Trizol (Thermo Fisher Scientific/Invitrogen). RNA was subsequently treated with 50 units/ml of RQ1 DNase (Promega) at 37°C for 1 h to eliminate traces of genomic DNA. First-strand cDNA synthesis (RT) was typically performed in 10 μl reactions containing 2.5 μg of total RNA, 50 pmol of a random decamer primer (N10), 40 units of rRNAsin (Promega) and 100 units of SuperScript III reverse transcriptase (Thermo Fisher Scientific/Invitrogen) at 50°C for 1 h. Regular PCRs were carried out using Taq DNA polymerase (KAPA Biosystems) and amplification products were resolved by gel electrophoresis in 2% agarose gels. Quantitative PCR (qPCR) assays were done in triplicate using SYBR^®^ FAST qPCR Master Mix (KAPA Biosystems) and a StepOnePlus real-time PCR system (Applied Biosystems) and the signals were normalized to Gapdh mRNA levels. All primer sequences are provided in Supplementary Table S4.

### *In vitro* RNA synthesis using T7 RNA polymerase

DNA templates were obtained by either linearizing T7 promoter-containing plasmids with appropriate restriction enzymes or by PCR amplification of gene fragments using forward primers containing a 5′-terminal T7 promoter overhang. Unlabeled RNA substrates were prepared by transcribing the DNAs in 25 μl reactions containing 30 units of T7 RNA polymerase, 10 mM DTT, 0.8 mM Ribo m^7^G Cap Analog, rNTP capping mix [0.5 mM rATP, 0.5 mM rCTP, 0.5 mM rUTP and 0.2 mM rGTP], 20 units of rRNAsin for 2 h at 37°C (all reagents from Promega). To generate radiolabeled RNA fragments, we replaced 0.48 mM rUTP with 20 μCi α^32^P-UTP (Perkin Elmer; NEG007X250UC). Biotin-labeled RNA baits were prepared by replacing the rNTP capping mix and Ribo m^7^G Cap Analog with the biotin RNA labeling mix (Roche). Reactions were stopped by adding 1 unit of RQ1 DNase (Promega) per 1 μg of template DNA and incubating the mixtures at 37°C for an additional 15 min. RNAs were then extracted using phenol-chloroform (1:1) mixture, precipitated with ethanol and rehydrated in DEPC-treated water.

### *In vitro* splicing assays

Splicing reactions were carried out as described (33). In some experiments, we used a nuclear extract immunodepleted for PTBP1. For this purpose, 40 μg of mouse monoclonal anti-PTBP1 antibody (Thermo Fisher Scientific/Invitrogen; clone 1) was incubated with 40 μl of protein G Sepharose beads (GE Healthcare) at 4°C overnight with continuous agitation. Beads were subsequently washed three times with buffer D (20 mM HEPES, pH 7.9, 100 mM KCl, 20% glycerol, 0.5 mM DTT and 0.2 mM EDTA) and incubated with 50 μl of HeLa S3 nuclear extract (pre-dialyzed against buffer D) for another 4 h at 4°C with agitation. The PTBP1-depleted nuclear extract was then recovered by pelleting the beads at 3000 rpm for 2 min.

### Pull-down assays

Pull-down assays were carried out by incubating 2 μg of biotinylated RNA baits in 400 μl of binding buffer (20 mM HEPES–KOH, pH 7.5, 100 mM KCl, 10 mM MgCl_2_, 0.01% Nonidet P-40 and 1 mM DTT) supplemented with 500 μg HeLa S3 nuclear extract (pre-dialyzed against buffer D) for 1 h at room temperature. RNA-protein complexes were then incubated with 30 μl of Streptavidin Sepharose Beads (Sigma) pre-washed in washing buffer (20 mM HEPES–KOH, pH 7.5, 200 mM KCl, 10 mM MgCl_2_, 0.01% Nonidet P-40 and 1 mM DTT) and pre-blocked with 0.2 mg/ml BSA for 1 h in 4°C. The beads were then washed three times with washing buffer and the RNA-associated proteins were eluted by boiling the beads for 10 min in 30 μl of 1 × SDS PAGE sample buffer (0.0625 M Tris–HCl pH 6.8, 2% SDS, 5% β-mercaptoethanol, 10% glycerol and 0.01% bromophenol blue) and subsequently analyzed by immunoblotting. To isolate the RNA fraction, beads were incubated with 250 μl of denaturation buffer (20 mM Tris–HCl, 4 M Urea, 0.5% SDS, 10 mM EDTA, 0.3 M NaCl) at 37°C for 10 min followed by phenol–chloroform extraction and RNA precipitation.

### RNase H protection assays

Forty thousand cpm of radiolabeled RNA was preincubated in 20 μl splicing mixtures for 15 min at 30°C, supplemented with 75 pmol of a DNA oligonucleotide complementary to the natural Dtx2 e6 5′ss (5′-CACAACTACCTTTT-3) or its 5′G4A variant (5′-CACAATTACCTTTT-3′) and 0.5 units of RNase H (NEB). The incubation was continued at 30°C for 15 min followed by phenol-chloroform extraction and RNA precipitation. In some samples, nuclear extract was pre-incubated with 75 pmol of the U1 antisense 2’OMe-RNA (5′-UGCCAGGUAAGUAU-3′) at 30°C for 10 min prior to adding labeled RNAs. Cleaved RNA products were separated by 6% denaturing polyacrylamide gel electrophoresis and visualized using a using a Typhoon Trio phosphorimager (GE Healthcare).

### Immunoblotting

Proteins were extracted from PBS-washed adherent cells using NP40 buffer [20 mM Tris–HCl, pH 7.5, 150 mM NaCl, 5 mM EDTA, 10% glycerol, 1% Nonidet P-40, 1 mM phenylmethanesulfonyl fluoride and cOmplete EDTA-free protease inhibitor cocktail (Roche; one tablet per 50 ml)] and quantified using a BCA protein assay kit (Thermo Scientific). Proteins were then separated by 4–20% gradient SDS-PAGE (Bio-Rad), electrotransferred to nitrocellulose membranes and analyzed using the following primary antibodies: mouse monoclonal anti-PTBP1 (1:1000; Thermo Fisher Scientific/Invitrogen), mouse monoclonal anti-PTBP2 (1:20 000; a gift from R. Darnell), mouse monoclonal anti-U1-70K (1:1000; a gift from T. Maniatis), mouse monoclonal anti-Gapdh (1:10 000; Thermo Fisher Scientific/Ambion). Immunoblot signals were visualized using corresponding secondary antibodies conjugated with horseradish peroxidase (GE Healthcare) and Immobilon Western Chemiluminescent HRP Substrate (EMD Millipore).

### Bioinformatics

CAD cells treated with siControl, siPtbp1 or siPtbp1/2 were analyzed by RNA-seq as described [(44); NCBI GEO accession number GSE37933]. Alternative splicing changes were quantified using ExpressionPlot (45) and cassette exons consistently up- or down-regulated in both siPtbp1 and siPtbp1/2 samples compared to the siControl (*P* < 0.01) were shortlisted for further analyses. Genes with >1 exon regulated in response to siPtbp1 or siPtbp2 were omitted. The 5′ss and 3′ss sequences were analyzed by MaxEntScan and WebLogo (46,47) using internal constitutive exons from PTBP1/2-regulated transcripts as controls. Exonic splicing enhancers and silencers were predicted using the FIMO program from the MEME suite (48) based on published position weight matrices (49–51). Matches with *P* < 0.005 were used for further analyses. To detect motif enrichment in intronic sequences preceding and following regulated exons, we computed GC-compensated average motif affinity (AMA) *P*-values for the PTBP1/2-specific position weight matrix [M227 from the CisBP-RNA database; (52)] using the MEME suite. Sequences with *P* < 0.05 following the Benjamini-Hochberg adjustment for multiple testing were considered enriched for PTBP1/2 interaction motifs.

## RESULTS

### Characteristic features of mouse alternative exons activated by PTBP1

In search for a mechanism that might allow PTBP1 to function as a bona fide splicing activator we examined cassette exons consistently up- and down-regulated upon knocking down this RBP alone (siPtbp1) or together with its functionally similar paralog PTBP2 (27) (siPtbp1/2) in the mouse neuroblastoma cell line CAD (Supplementary Tables S1 and S2; see Materials and Methods). In line with published analyses for human cells (28,29), inspection of our RNA-seq data showed relative enrichment of PTBP1/2-specific interaction motifs upstream of the PTBP1-repressed and downstream of the PTBP1-activated exons (Supplementary Figure S1A and see Materials and Methods).

PTBP1-repressed exons tended to be smaller than [median length 61.5 nt; two-sided Kolmogorov–Smirnov (KS) test *P*-value 1.1 × 10^−16^] while PTBP1-activated exons were statistically indistinguishable (median 126 nt; KS test *P*-value 0.78) from their constitutive counterparts (median 120 nt). All regulated exons had relatively weak 5′ and 3′ splice sites (ss) but the 5′ss scores of the activated exons were significantly lower compared to the repressed ones (*P* = 7.4 × 10^−3^; Supplementary Figure S1B and C). Consistent with this observation, the 5′ss sequences of activated exons often lacked the canonical AGU suffix in the MAG|GURAGU consensus sequence known to base pair with the 5′ end of the spliceosomal snRNA U1 [(53); Supplementary Figure S1D]. We also analyzed densities of exonic splicing enhancer (ESE) and exonic splicing silencer (ESS) motifs but did not detect any differences among the corresponding regulated and non-regulated categories (Supplementary Figure S2).

The bioinformatically predicted role of PTBP1 in splicing activation was confirmed for 10 alternative exons that were shortlisted for RT-PCR validation (Supplementary Figure S3; also see Dtx2 data below). All these genes had discernable PTBP1/2-specific motifs downstream of the regulated exons (Supplementary Figure S3 and see below). Overall, these data point at the possibility that activation of mouse alternative exons by PTBP1 might depend on its recruitment downstream of a suboptimal 5’ss.

### Ptbp1 promotes inclusion of the alternative exon 6 in Dtx2 mRNA

**Figure 1.**
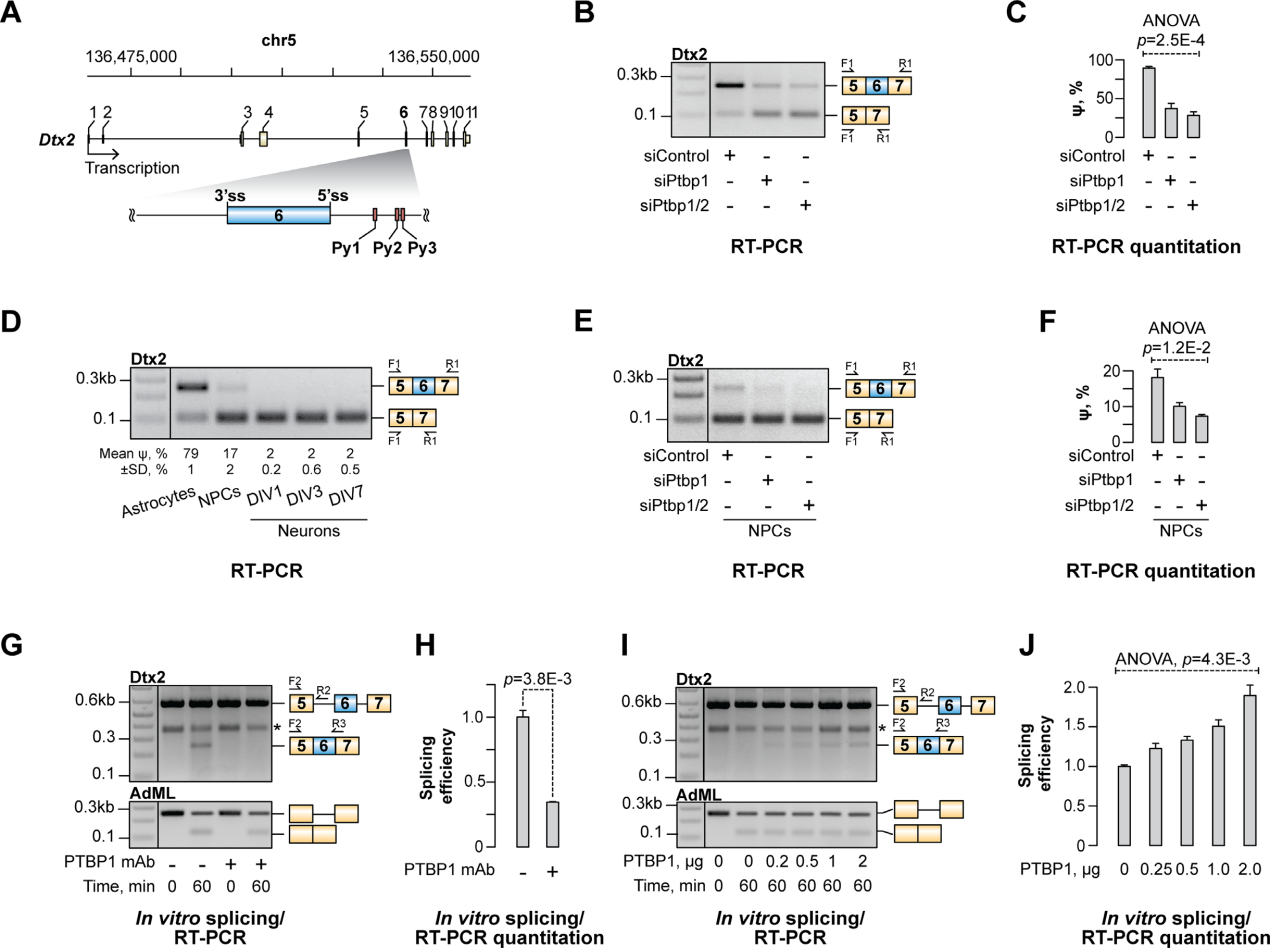
PTBP1 stimulates inclusion of Dtx2 exon 6. (**A**) A diagram of the mouse *Dtx2* gene showing the PTBP1-stimulated exon 6 in its immediate intronic context. Py1, Py2 and Py3 are downstream elements containing PTBP1 interaction motifs. (**B**) CAD cells were treated with a PTBP1-specific siRNA (siPtbp1), a mixture of siPtbp1 and PTBP2-specific siRNA (siPtbp2) or a control non-targeting siRNA (siControl) and the inclusion of the PTBP1-activated exon 6 (e6) into Dtx2 transcripts was analyzed by RT-PCR. Note that siPtbp1 and siPtbp2 promote e6 skipping. (**C**) Quantification of the e6-specific ‘percent spliced in’ (ψ) values in (B). (**D**) An RT-PCR assay showing that Dtx2 e6 is more efficiently included in astrocytes and NPCs than in neurons. The DIV labels indicate the number of days primary neuronal cultures were maintained *in vitro*. (**E**) NPCs were treated with siRNAs as in (B) and the changes in the Dtx2 splicing pattern were analyzed by RT-PCR. (**F**) Quantification of the data in (E) showing a significant decrease in e6 inclusion efficiency in response to siPtbp1 and siPtbp1/2. (**G**) Splicing of the Dtx2 or the adenovirus major late (AdML) control RNA substrates was assayed *in vitro* using either control-treated or PTBP1-depleted nuclear extracts and the reaction products were analyzed by RT-PCR at 0- and 60-min time points. Note that depletion of PTBP1 inhibits e6 inclusion into Dtx2-derived products but has no effect on the efficiency of AdML splicing. The asterisk marks an unspecific RT-PCR band visible at both 0 and 60 min. The Dtx2 RNA substrate was produced by *in vitro* transcription of the DNA fragment amplified with the EMO4721 and EMO4722 primers (Supplementary Table S4). (**H**) Difference in the abundance of the e6-containing products between the control and the PTBP1-depleted 60-min samples quantified from (G). The value in the control sample is set to 1. (**I**) PTBP1-depleted splicing reactions were supplemented with indicated amounts of purified recombinant PTBP1 protein and the reaction products were analyzed as in (G). (**J**) Quantification of the e6-containing reaction product in (I). The value in the PTBP1-depleted sample is set to 1. Quantitative data in (C, D, F, H and J) are averaged from three independent experiments ± SD. One-way ANOVA was used in (C, F and J) and a two-tailed *t*-test assuming unequal variances in (H).

To test this prediction, we turned to the PTBP1-activated exon 6 (e6) of the *Dtx2* gene encoding a regulator of the Notch pathway (54,55) (Figure 1A). This exon has a characteristic for its class length (138 nt; comparable to the 126 nt median) and a suboptimal 5’ss (AAG|GUAGUU) followed by putative PTBP1 interaction sites (Figure 1A). We validated PTBP1 dependence of this exon by treating CAD cells with a control siRNA (siControl), siPtbp1 or siPtbp1/2 and analyzing changes in e6 inclusion by RT-PCR (Figure 1B and Supplementary Figure S4A). Control-treated cells expressed predominantly the e6-containing form of the Dtx2 mRNA (Figure 1B and C; ‘percent spliced in’ value ψ = 89%), whereas depletion of PTBP1 alone or together with PTBP2 reduced e6 inclusion to 37% and 28%, respectively (ANOVA p-value 2.54 × 10^−4^). e6 splicing was unaffected in cells treated with siPtbp2 only (Supplementary Figure S4B), consistent with the relatively low expression of PTBP2 protein in in the presence of PTBP1 (44,56). Of note, the siRNAs used in our experiments had no effect on the overall Dtx2 mRNA levels (Supplementary Figure S4C).

Ptbp1 is naturally down-regulated by the microRNA miR-124 in developing neurons, but not in astrocytes (56,57). We therefore wondered whether Dtx2 e6 was differentially spliced in the corresponding cell types. RT-PCR assays showed that mouse cortical astrocytes expressed predominantly e6-included Dtx2 transcripts (ψ = 79%; Figure 1D). On the other hand, this form was noticeably less abundant in cortical neuronal progenitor cells (NPCs; ψ = 17%) and virtually absent in cortical neurons (ψ = 2%; Figure 1D). RT-qPCR using PTBP1-specific primers showed a strong positive correlation between PTBP1 expression levels and e6 inclusion (Pearson's correlation coefficient ρ = 0.98, *P* = 7.0 × 10^−7^; Supplementary Figure S4D). Importantly, treating NPCs with siPtbp1 or siPtbp1/2 reduced the e6 inclusion efficiency 1.8 and 2.5 times, respectively (ANOVA p value = 1.20 × 10^−2^; Figure 1E and F and Supplementary Figure S4E).

To confirm the role of PTBP1 in e6 activation, we assayed splicing of the Dtx2 pre-mRNA fragment spanning exons e5, e6 and e7 and the two intervening introns in HeLa nuclear extract (NE) known to contain a substantial amount of PTBP1 [Figure 1G and H; (33)]. A splicing product containing e6 was readily detectable after 1-hour incubation at 30°C. However, when we repeated the analysis using a PTBP1-immunodepleted extract, this product failed to form (Figure 1G and H). The inclusion of e6 was rescued by the addition of recombinant PTBP1 to the immunodepleted NE (Figure 1I and J). We concluded that PTBP1 is essential for optimal inclusion of the Dtx2 e6 exon.

### Inclusion of e6 depends on downstream pyrimidine-rich intronic elements

**Figure 2.**
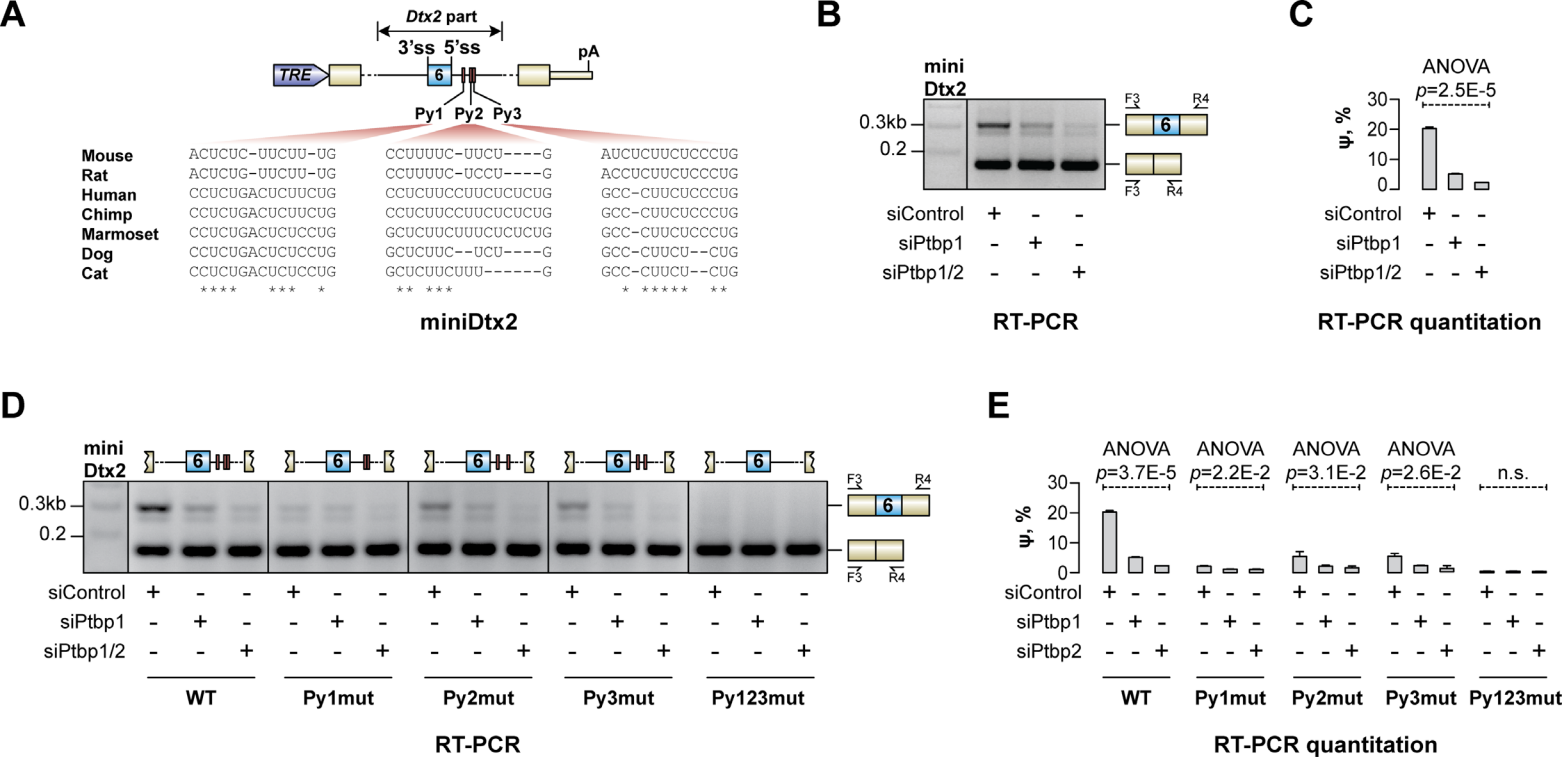
Inclusion of e6 requires downstream pyrimidine-rich sequences. (**A**) TRE promoter-driven minigene construct (miniDtx2) with the Dtx2 e6 and its flanking intronic sequences embedded within a recombinant exon-intron-exon unit. Downstream intronic pyrimidine-rich sequences (Py1–3) are depicted as red boxes and their sequences in mouse and several other mammalian species are shown below. (**B**) CAD cells pretreated with the siControl, siPtbp1 or siPtbp1/2 were transfected with miniDtx2 and the minigene-specific splicing patterns were analyzed by RT-PCR using the F3/R4 primer pair. Note that the e6-containing minigene transcripts are readily detectable in the siControl sample but are progressively down-regulated upon PTBP1 and PTBP2 knockdown. (**C**) Quantitation of e6 inclusion in (B). (**D**) CAD cells pretreated with the indicated siRNAs were transfected with either the wild-type (WT) miniDtx2 construct or its derivatives with mutated Py elements (Py1mut, Py2mut, Py3mut or Py123mut, respectively) and analyzed by RT-PCR as in (B). Note that the loss of individual Py elements reduces e6 inclusion (especially evident in the siControl samples) and simultaneous inactivation of all three Py's renders the e6-containing splice form virtually undetectable. (**E**) Quantitation of e6-specific ψ values in (D). The ψ values in (C) and (E) are averaged from three independent experiments ± SD and compared using one-way ANOVA.

To find out whether PTBP1 can function as a bona fide splicing activator, we prepared a doxycycline-inducible miniDtx2 minigene construct where the Dtx2 e6 in its natural intronic context was integrated into a recombinant constitutive intron (Figure 2A). CAD cells pretreated with siControl, siPtbp1 or siPtbp1/2 were transfected with miniDtx2 and the minigene-specific splicing patterns were analyzed using RT-PCR with F3/R4 primers (Figure 2B and C). Regulation of the miniDtx2 transcripts was similar to that of the endogenous Dtx2 pre-mRNAs, with the e6 inclusion decreasing from ψ = 20% in the control to 5% and 2% in the siPtbp1- and Ptbp1/2- treated samples (Figure 2B and C).

We mapped *cis*-elements required for e6 inclusion by pretreating CAD cells with siControl, siPtbp1 or siPtbp1/2 and transfecting them with miniDtx2 variants lacking distal parts of the Dtx2-derived downstream intron (Supplementary Figure S5A). Notably, deletion of 210 nt- or 250 nt-long intronic sequences dramatically reduced e6 inclusion (Supplementary Figure S5B-C; compare the siControl samples). On the other hand, minigene lacking only a 150 nt-long intronic sequence behaved similarly to the full-length miniDtx2 suggesting that the 50–150 nt sequence window downstream of the e6 was required for optimal e6 inclusion.

This intronic region contains three evolutionarily conserved blocks of consensus PTBP1-specific motifs (Py1, Py2 and Py3; Figure 2A). To address possible contribution of these elements to e6 splicing, we transfected siRNA-treated CAD cells with full-length miniDtx2 minigenes containing corresponding site-specific mutations (Figure 2D). RT-PCR analyses showed that mutating individual Py's reduced e6 inclusion with the Py1mut showing the strongest effect. Combined inactivation of all three Py's (Py123mut) led to a complete skipping of e6 even in the siControl samples (Figure 2D–E).

To test whether PTBP1 directly interacted with the corresponding part of the Dtx2 pre-mRNA, we prepared biotinylated RNA fragments containing either the wild-type (WT) or mutant Py1, Py2 and Py3 sequences and analyzed recruitment of PTBP1 protein to these ‘baits’ in HeLa NE using a streptavidin pull-down assay (Supplementary Figure S6). Immunoblot analyses of the bait-associated material revealed relatively efficient interaction of PTBP1 with the WT probe. Notably, PTBP1 binding was significantly reduced by inactivating individual Py's and virtually abolished when the three elements were mutated simultaneously (Supplementary Figure S6). These data suggest that PTBP1 may promote e6 inclusion by interacting with the Py elements in the downstream intron.

### Suboptimal 5′ splicing site is critical for e6 regulation

**Figure 3.**
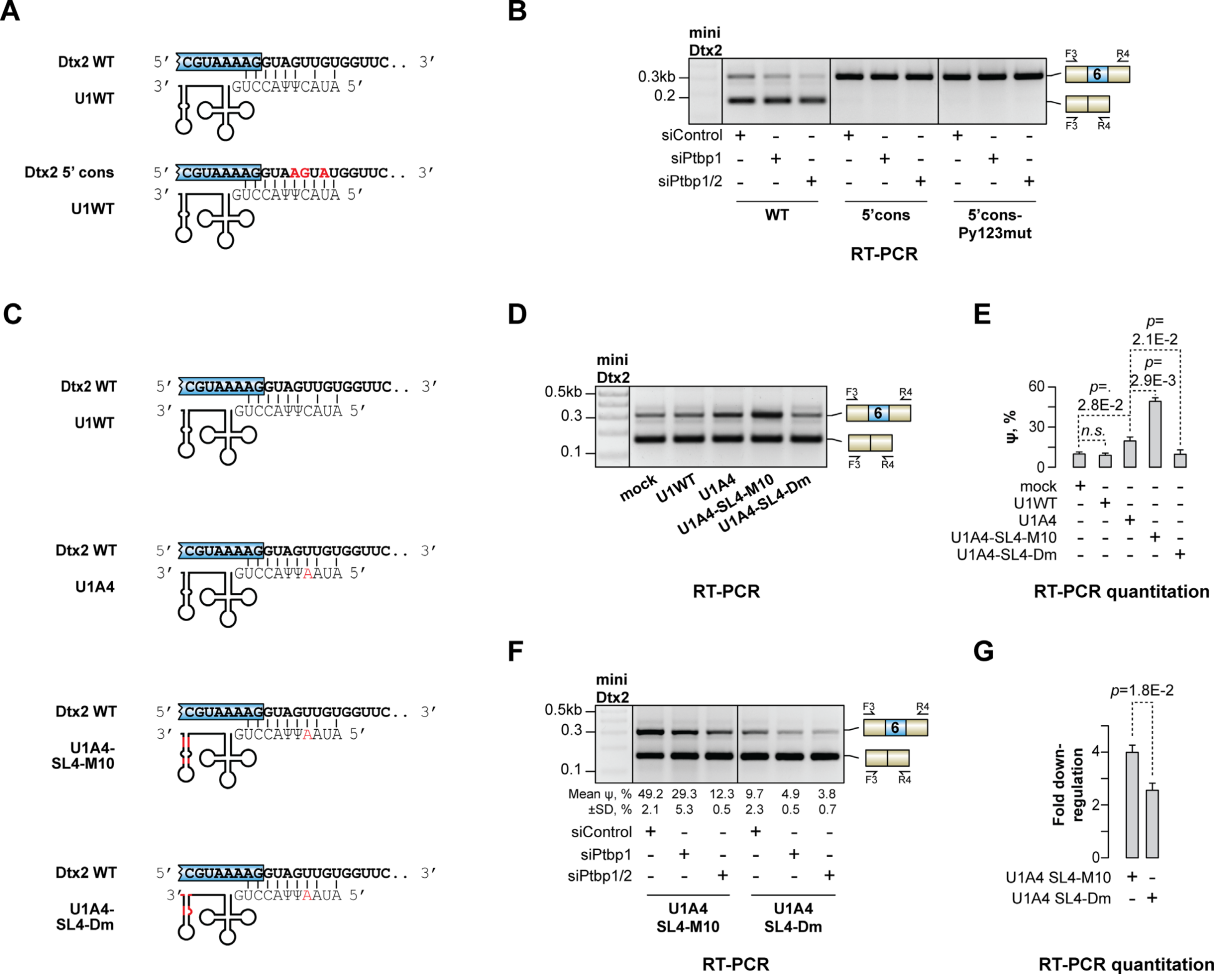
Inclusion of e6 depends on interaction between PTBP1 and U1. (**A**) Base pairing between cell-encoded U1 snRNA and either the wild-type e6 5’ss (miniDtx2-WT) or its genetically improved version matching the 5’ss consensus sequence (miniDtx2–5’cons). Mutated miniDtx2 nucleotides are shown in red. (**B**) CAD cells pretreated with siControl, siPtbp1 or siPtbp1/2 were transfected with miniDtx2-WT, miniDtx2–5’cons or miniDtx2–5’cons-Py123mut (a derivative of miniDtx2–5’cons additionally lacking the three downstream Py elements) and the minigene-specific splicing patterns were analyzed by RT-PCR with the F3/R4 primers. Note that 5’cons transforms e6 into a constitutive exon included regardless of the PTBP1 and PTBP2 levels and the presence of the Py's. (**C**) Base pairing between the miniDtx2-WT transcripts and four recombinant U1 snRNAs: a wild-type version (U1WT), its C4A-mutant (U1A4) and two U1A4 derivatives with modifications in the SL4 stem-loop (U1A4-SL4-M10 and U1A4-SL4-Dm) predicted to strengthen and weaken PTBP1 binding, respectively. The modified parts of U1 are shown in red. (**D**) CAD cells were co-transfected with the miniDtx2-WT minigene and either an expression construct encoding a recombinant U1 snRNA (U1WT, U1A4, U1A4-SL4-M10 or U1A4-SL4-Dm) or the corresponding empty vector (mock) and analyzed by RT-PCR as in (B). Note that U1A4 promotes e6 inclusion compared to U1WT and the mock. The addition of the SL4-M10 mutation to the U1A4 background enhances this stimulatory effect whereas SL4-Dm reduces it significantly. (**E**) e6 ψ values quantified from (D). (**F**) CAD cells pretreated with the indicated siRNAs were co-transfected with miniDtx2-WT and either U1A4-SL4-M10 or U1A4-SL4-Dm and analyzed by RT-PCR as in (B). (**G**) The repressive effect of PTBP1/2 knockdown on e6 inclusion was calculated by comparing the ψ values between the corresponding siPtbp1/2 and siControl samples in (F) as fold down-regulation. Note that e6 is more sensitive to PTBP1/2 levels in the case of U1A4-SL4-M10 compared to U1A4-SL4-Dm, consistent with the SL4 properties in these two snRNAs. Quantitative data in (E-G) are averaged from three independent experiments ±SD. Samples in (E and G) are compared by a two-tailed t-test assuming unequal variances.

To understand its possible functional significance, we changed the relatively weak 5′ss of e6 to the consensus 5’ss (5’cons) thus effectively extending its base pairing with the U1 snRNA (Figure 3A). When introduced into the miniDtx2 minigene, this mutation transformed e6 into a constitutive exon that was efficiently included regardless of the PTBP1 and the PTBP2 expression levels (Figure 3B). Similar constitutive splicing pattern was observed when we combined the 5’cons mutation with inactivation of all three downstream Py sequences (Py123mut) (Figure 3B).

In a reciprocal experiment, we co-transfected the wild-type miniDtx2 with the U1A4 snRNA expression construct containing a C4A substitution that was predicted to improve U1 base pairing with the natural e6 5′ss (Figure 3C and Supplementary Figure S7A). This stimulated e6 inclusion significantly compared to an empty vector or an expression construct encoding a wild-type U1 snRNA (Figure 3D and E). The increase in the relative abundance of the e6-containing splice form in the U1A4 samples was less pronounced than in the case of miniDtx2–5′cons, consistent with some mismatches remaining in the U1A4/5′ss duplex (Figure 3C). Thus, a suboptimal interaction between U1 snRNA and the 5′ss of e6 is critical for proper regulation of this exon by PTBP1.

### Activation of e6 depends on interaction between PTBP1 and U1

Ptbp1 has been shown to contact the U1 stem-loop 4 (SL4) in the context of splicing repression (58). This hinders the assembly of a functional spliceosome on the SRC/c-src pre-mRNA giving rise to non-productive RNP complexes (58). We wondered if in the case of the Dtx2 pre-mRNA the interaction between PTBP1 and U1 snRNP could stimulate e6 inclusion by promoting U1 recruitment to the weak 5’ss. To examine this possibility, we modulated the strength of the U1-PTBP1 interaction by introducing corresponding changes to the SL4 element in the U1A4 suppressor. The interaction was strengthened in the U1A4-SL4-M10 construct (Figure 3C) by substituting several G/C base pairs in the SL4 stem with A/Us (59). Conversely, the U1A4-SL4-Dm construct (Figure 3C) contained the *Drosophila melanogaster* SL4 interacting with PTBP1 less efficiently than its mammalian counterpart (59). Gratifyingly, co-expressing U1A4-SL4-M10 with the wild-type miniDtx2 minigene significantly promoted and U1A4-SL4-Dm reduced e6 inclusion e6 inclusion compared to U1A4 (Figure 3D and E). This effect relied on the presence of the downstream Py elements since we did not detect any difference in the minute amounts of the e6-containing isoform produced from Py123mut miniDtx2 co-transfected with the U1A4, U1A4-SL4-M10 and U1A4-SL4-Dm (Supplementary Figure S7B and C).

Of note, the inclusion of e6 into the wild-type miniDtx2 transcripts was less responsive to PTBP1/2 expression levels in the presence of U1A4-SL4-Dm as compared to U1A4-SL4-M10 (Figure 3F–G). Simultaneous knockdown of PTBP1 and PTBP2 led to a ∼4.0-fold decrease in e6 inclusion in the U1A4-SL4-M10 samples and only a ∼2.5-fold decrease in the U1A4-SL4-Dm samples (*t*-test, *P* = 0.018; Figure 3G). This difference depended on the Py's since co-transfection of U1A4-SL4-M10 or U1A4-SL4-Dm with miniDtx2-Py123mut gave rise to indistinguishable splicing patterns (Supplementary Figure S7D-F). These results indicated that PTBP1 may promote U1 recruitment to a suboptimal 5′ss in a splicing activation context.

### Ptbp1 promotes U1 recruitment to the e6 5’ss

**Figure 4.**
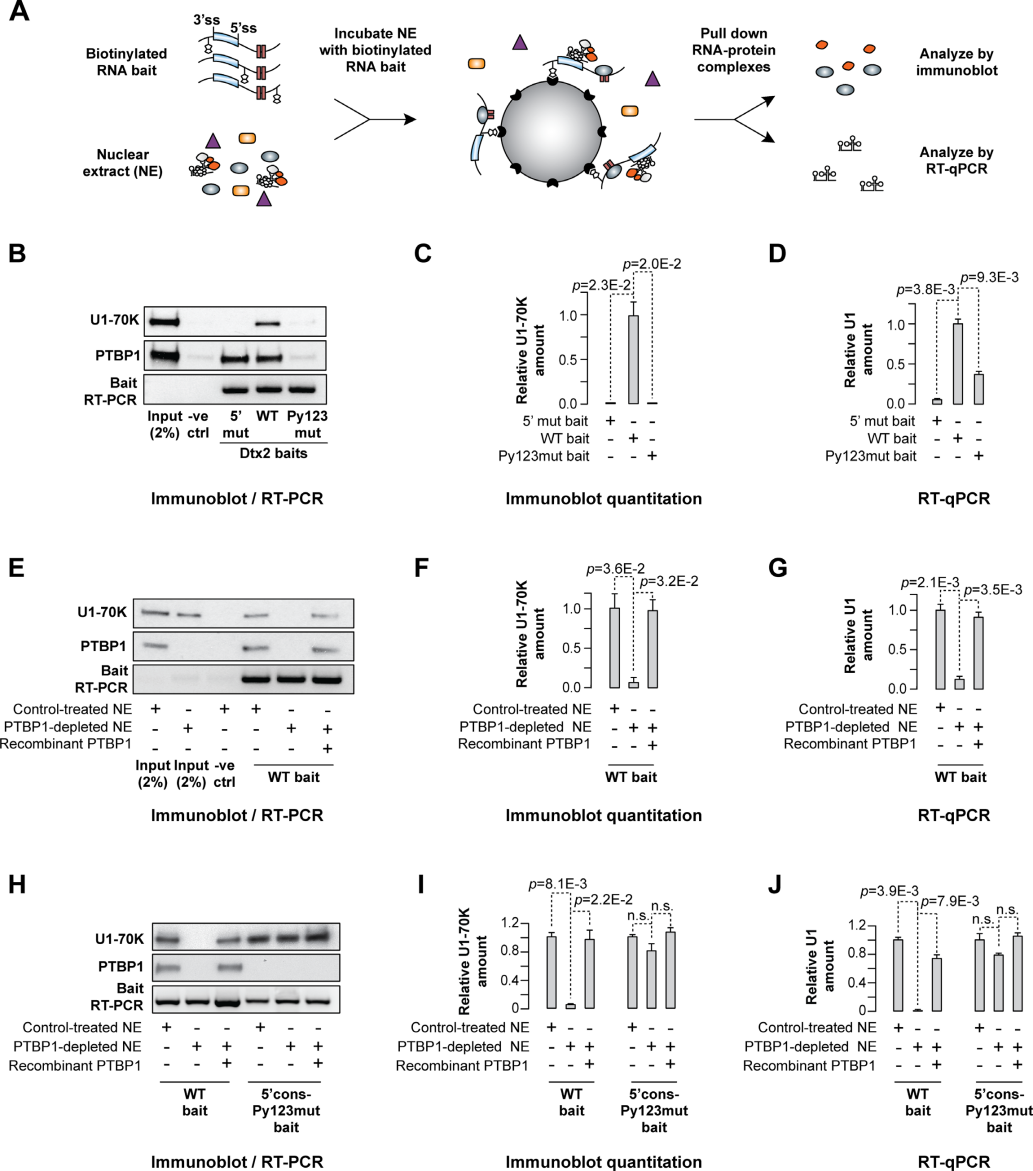
Recruitment of U1 snRNP to Dtx2 pre-mRNA depends on PTBP1. (**A**) A diagram of the pull-down assay. (**B–D**) Biotinylated RNA baits with the Dtx2 e6 either in the wild-type intronic context (WT) or containing loss-of-function mutations of the 5’ss (5′mut) or the three Py elements (Py123mut) were prepared by *in vitro* transcription of corresponding DNA fragments amplified with the EMO4983 and EMO4782 primers (Supplementary Table S4). Following a 1-hour incubation with HeLa nuclear, the recruitment of the U1 snRNP and the PTBP1 protein to the baits was analyzed by (B and C) immunoblotting with U1-70K and PTBP1-specific antibodies or (D) RT-qPCR with U1 snRNA-specific primers. (B) Unlabeled WT Dtx2 RNA was used as a negative control (-ve ctrl) and lane loading was estimated by the bait-specific RT-PCR. (C) U1-70K recruitment efficiency to the Dtx2 baits in (B) quantified by band densitometry. Note that association of U1 snRNP with the bait requires PTBP1 binding but not vice versa. (**E–G**) Biotinylated WT Dtx2 RNA was incubated with either control-treated or PTBP1-immunodepeleted nuclear extract with or without the addition of purified recombinant PTBP1 protein (2 μg per 250 μg of NE) and the recruitment of U1 snRNP and PTBP1 was analyzed as in (B–D). Note that U1 fails to form a stable complex with the bait in the PTBP1-depleted extract and that recombinant PTBP1 rescues this effect. (**H–J**) Comparison of the U1 snRNP and the PTBP1 binding properties between the WT bait and its derivative containing the consensus 5’ss and no Py elements (5’cons-Py123mut) carried out as described in (B–D). Note that U1 snRNP interacts with 5’cons-Py123mut in a PTBP1-independent manner, as expected. Quantitative data in (C, D, F, G, I and J) are averaged from three independent experiments ± SD and compared by a two-tailed *t*-test assuming unequal variances.

As a direct test of this model, we incubated a biotinylated RNA fragment containing Dtx2 e6 in its natural intronic context (WT) with HeLa NE, and purified factors interacting with this ‘bait’ using streptavidin beads (Figure 4A). Immunoblot analyses of the bait-associated material showed that the WT bait efficiently recruited PTBP1 and an integral U1 snRNP component, U1-70K (Figure 4B and C). U1-70K failed to interact with a 5’ss-inactivated (5’mut) version of the bait while PTBP1 binding to this RNA molecule was not affected. Importantly, mutations of the downstream Py elements diminished recruitment of PTBP1 and U1–70K in a concomitant manner (Figure 4B–C and Supplementary Figure S8). U1-70K was an adequate proxy for the entire U1 snRNP complex since RT-qPCR using U1 snRNA-specific primers detected similar differences in interaction efficiencies among the three baits (Figure 4D).

The U1 snRNP recruitment to the WT bait required PTBP1 protein since incubating the wild-type bait with a PTBP1-immunodepleted extract significantly decreased U1-70K and U1 snRNA pull-down efficiencies (Figure 4E–G). The addition of purified recombinant PTBP1 back to the immunodepleted extracts rescued U1 snRNP binding completely (Figure 4E–G). As expected, U1 snRNP binding to the 5’cons-Py123mut bait containing the consensus 5’ss and no Py's did not depend on PTBP1 (Figure 4H–J).

**Figure 5.**
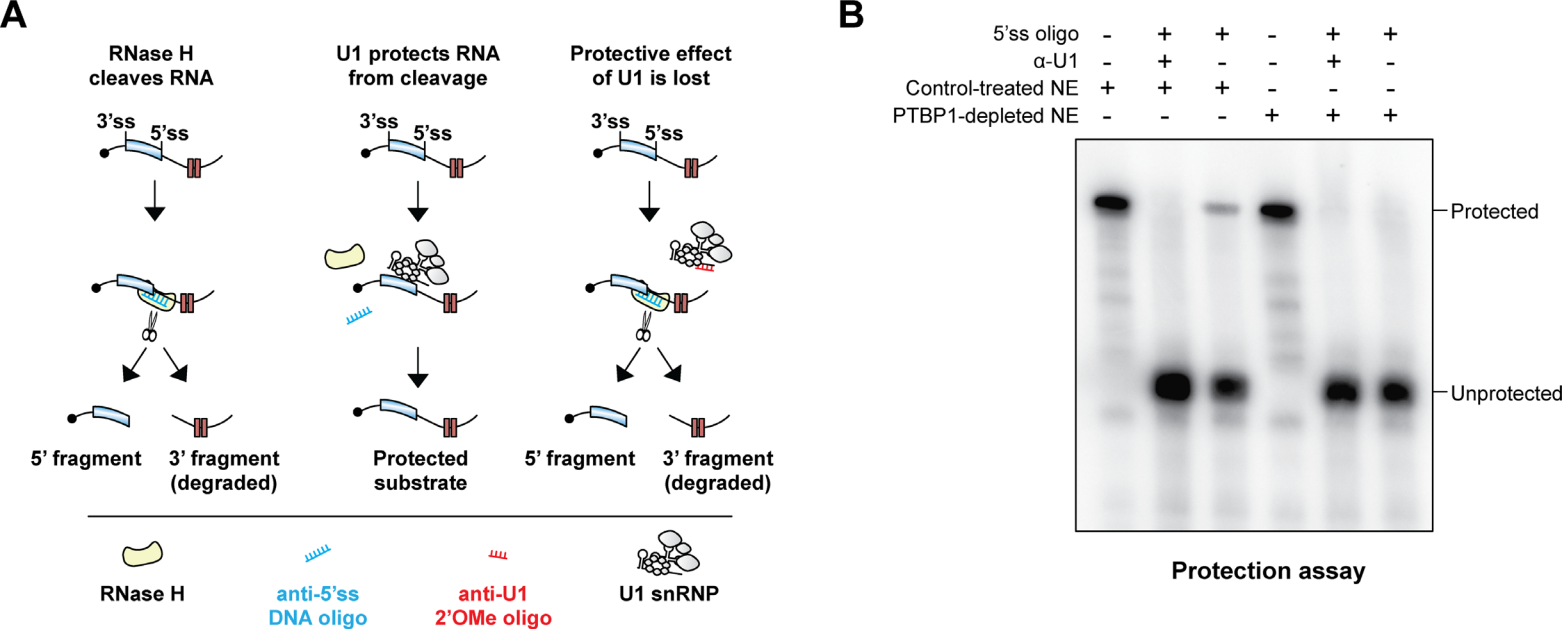
PTBP1 recruits U1 snRNP to the e6 5’ss. (**A**) Diagram of the RNase H protection assay. (**B**) ^32^P-labeled WT probes designed as in Figure 4 were incubated with either control-treated or PTBP1-depleted NE, the indicated oligonucleotides, and RNase H and the reaction products were analyzed by polyacrylamide gel electrophoresis under denaturing conditions. Note that a U1 snRNP-specific protection signal is present in the control but PTBP1-depleted samples. Only the 5′-terminal ‘unprotected’ RNA fragment is visible in this experiment possibly because it is capped and therefore more stable than the 3-terminal fragment.

We used an RNase H protection assay (Figure 5A) to ensure that U1 snRNP formed proper contacts with the e6 5’ss in the above experiments. The addition of RNase H and a 5’ss-complementary DNA oligonucleotide induced site-specific cleavage of the WT RNA pre-incubated with a control-treated HeLa NE; however, a readily detectable fraction of the substrate remained intact (Figure 5B). This was due to protection of the 5’ss by U1 snRNP since the uncut species disappeared when we repeated the experiment in the presence of a 2’OMe oligonucleotide complementary to the 5’ss-interacting U1 sequence. Notably, virtually no protection was observed when the experiment was repeated using a PTBP1-depleted NE (Figure 5B) or when the WT bait was substituted with its Py123mut counterpart (Supplementary Figure S9). Together, these data suggested that PTBP1 promotes physical recruitment of U1 snRNA to the e6 5’ss.

### A context-specific mechanism controls the choice between splicing activation and repression

Why does interaction between PTBP1 with U1 stimulate Dtx2 e6 while promoting the assembly of a non-productive splicing complex on the SRC pre-mRNA (58)? In the latter case, PTBP1 is known to interact with intronic pyrimidine-rich elements located both upstream and downstream of the regulated exon N1 (60). On the other hand, there are 11 consensus PTBP1 binding motifs (YUCUUY, YUCUCY, YUUCUY and YCUCUY) in a 125 nt intronic window following Dtx2 e6 and none in the similarly sized sequence preceding this exon (Figures 1A and 2A). Thus, the opposite outcomes of PTBP1-dependent U1 recruitment in these two systems might depend on whether PTBP1 binds both upstream and downstream or downstream only, consistent with an earlier observation (28).

**Figure 6.**
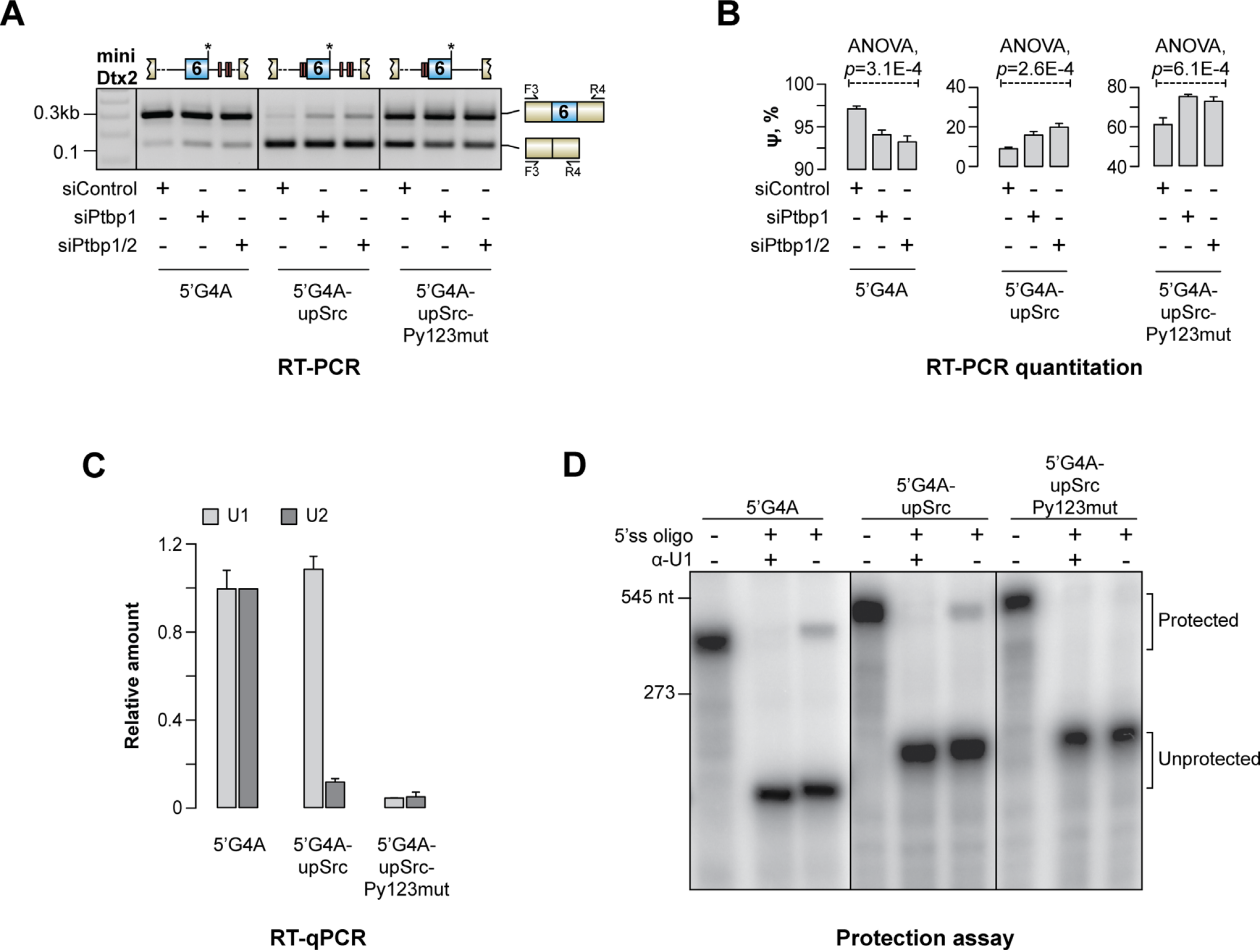
PTBP1 binding both upstream and downstream of e6 leads to a strong repression effect accompanied by the assembly of a splicing-deficient ribonucleoprotein complex. (**A**) CAD cells pretreated with siControl, siPtbp1 or siPtbp1/2 siRNAs were transfected with 5′G4A-modified miniDtx2 minigenes showing a more efficient inclusion of e6 compared to the miniDtx2-WT but (unlike miniDtx2–5′cons in Figure 3B) remaining responsive to the PTBP1 and PTBP2 levels. Three such minigenes were used: miniDtx2–5′G4A containing no additional changes, miniDtx2–5′G4A-upSrc containing an upstream PTBP1-interacting intronic sequence from the human *SRC* gene, and miniDtx2–5′G4A-upSrc-Py123mut containing the upstream *SRC* sequence but no downstream *Dtx2* Py elements. Analysis of the minigene-specific splicing patterns by RT-PCR with the F3/R4 primer pair showed that PTBP1 and PTBP2 stimulate e6 inclusion in the miniDtx2–5′G4A transcripts and that this effect is inverted for miniDtx2–5′G4A-upSrc and miniDtx2–5′G4A-upSrc-Py123mut. Importantly, e6 is repressed in the miniDtx2–5′G4A-upSrc context to a substantially greater extent than in miniDtx2–5′G4A-upSrc-Py123mut, which is especially obvious in the siControl samples. (**B**) Quantitation of e6 inclusion efficiency in (A). (**C**) Recruitment of U1 and U2 snRNPs to biotinylated RNA baits designed as explained in Figure 4 and matching the minigene sequences in (A-B) was analyzed in the corresponding pull-down fractions by RT-qPCR with snRNA-specific primer pairs. Note that miniDtx2–5′G4A interacts well with both U1 and U2 snRNPs whereas miniDtx2–5′G4A-upSrc-Py123mut recruits these snRNPs inefficiently. The addition of the upSrc element to miniDtx2–5′G4A diminishes its ability to recruit U2 but does not compromise U1 binding. Quantitative data in (B-C) are averaged from 3 independent experiments ± SD. (**D**) RNase H protection assay confirming that miniDtx2–5′G4A and miniDtx2-5′G4A-upSrc but not miniDtx2–5′G4A-upSrc-Py123mut allow U1 to form a stable complex with the e6 5’ss.

To address this possibility, we replaced the natural intronic sequence immediately preceding Dtx2 e6 with the corresponding SRC sequence previously shown to recruit PTBP1 (36,60). When we introduced this modification to the wild-type miniDtx2 minigene e6 became constitutively skipped regardless of the PTBP1 and PTBP2 expression levels (not shown). To facilitate further analyses, we slightly enhanced the natural e6 5’ss by substituting the G at the +4 position with an A. This improved base pairing between the 5’ss and the wild-type U1 snRNA, albeit to a level that was still suboptimal compared to the 5’ss consensus. Indeed, the modified minigene (miniDtx2–5’G4A) retained the e6 dependence on PTBP1 (Figure 6A and B).

Notably, swapping the natural upstream intronic sequence for its SRC N1 counterpart in this background (miniDtx2–5’G4A-upSrc) converted e6 into a PTBP1-repressible exon (Figure 6A and B and Supplementary Figure S11A). The SRC-derived sequence was sufficient for PTBP1-dependent repression in the absence of the Dtx2-specific downstream Py elements (Figure 6A and B). However, the overall extent of e6 repression was substantially lower in this case (miniDtx2-5’G4A-upSrc-Py123mut) than for miniDtx2-5’G4A-upSrc (Supplementary Figure S10). Similar results were obtained when we inserted the SRC PTBP1-binding sequence in front of the e6-specific branch point sequence and kept the natural Dtx2 3’ss region intact (miniDtx2-5’G4A-up2Src and miniDtx2–5’G4A-up2Src-Py123mut minigenes; Supplementary Figure S11A–C).

To understand the mechanism underlying the switch from activation to repression, we examined recruitment of U1 and U2 snRNPs to biotinylated RNA probes containing e6 in the 5’G4A, 5’G4A-upSrc or 5’G4A-upSrc-Py123mut contexts corresponding to the above minigenes. U1 interacted with the 5’G4A and 5’G4A-upSrc RNAs comparably well arguing that PTBP1 binding upstream of e6 does not interfere with this step of the spliceosome assembly (Figure 6C). In both cases U1 was recruited to its correct 5’ss position (Figure 6D). U1 recruitment to 5’G4A-upSrc-Py123mut was substantially less efficient (Figure 6C and D). On the other hand, U2 interacted well only with the 5’G4A but not the 5’G4A-upSrc or the 5’G4A-upSrc-Py123mut RNAs (Figure 6C). Similar U1 and U2 recruitment patterns were detected for the up2Src transcripts (Supplementary Figure S11D). Thus, recruitment of PTBP1 both upstream and downstream of e6 leads to a strong repression effect accompanied by the assembly of a splicing-deficient exonic complex containing U1 but no U2 snRNP.

### PTBP1-facilitated recruitment of U1 to the 5’ss is a general phenomenon

We finally asked whether facilitated recruitment of the U1 snRNP to the 5’ss could be a common mechanism for PTBP1-stimulated exon inclusion. To this end, we replaced most of the Dtx2 e6 with a SRC N1 sequence in the miniDtx2-5’G4A background (Supplementary Figure S12A) and assayed PTBP1 dependence of the resultant miniDtx2(SrcN1)-G4A minigene in CAD cells. Consistent with the presence Dtx2-specific intronic sequences in this construct, the inclusion of the recombinant exon was stimulated by PTBP1 (Supplementary Figure S12B). Our additional pull-down assays showed that PTBP1 was also required for efficient U1 recruitment to the biotinylated RNA bait corresponding to miniDtx2(SrcN1)-G4A and containing the recombinant exon in in its immediate intronic context (Supplementary Figure S12C).

**Figure 7.**
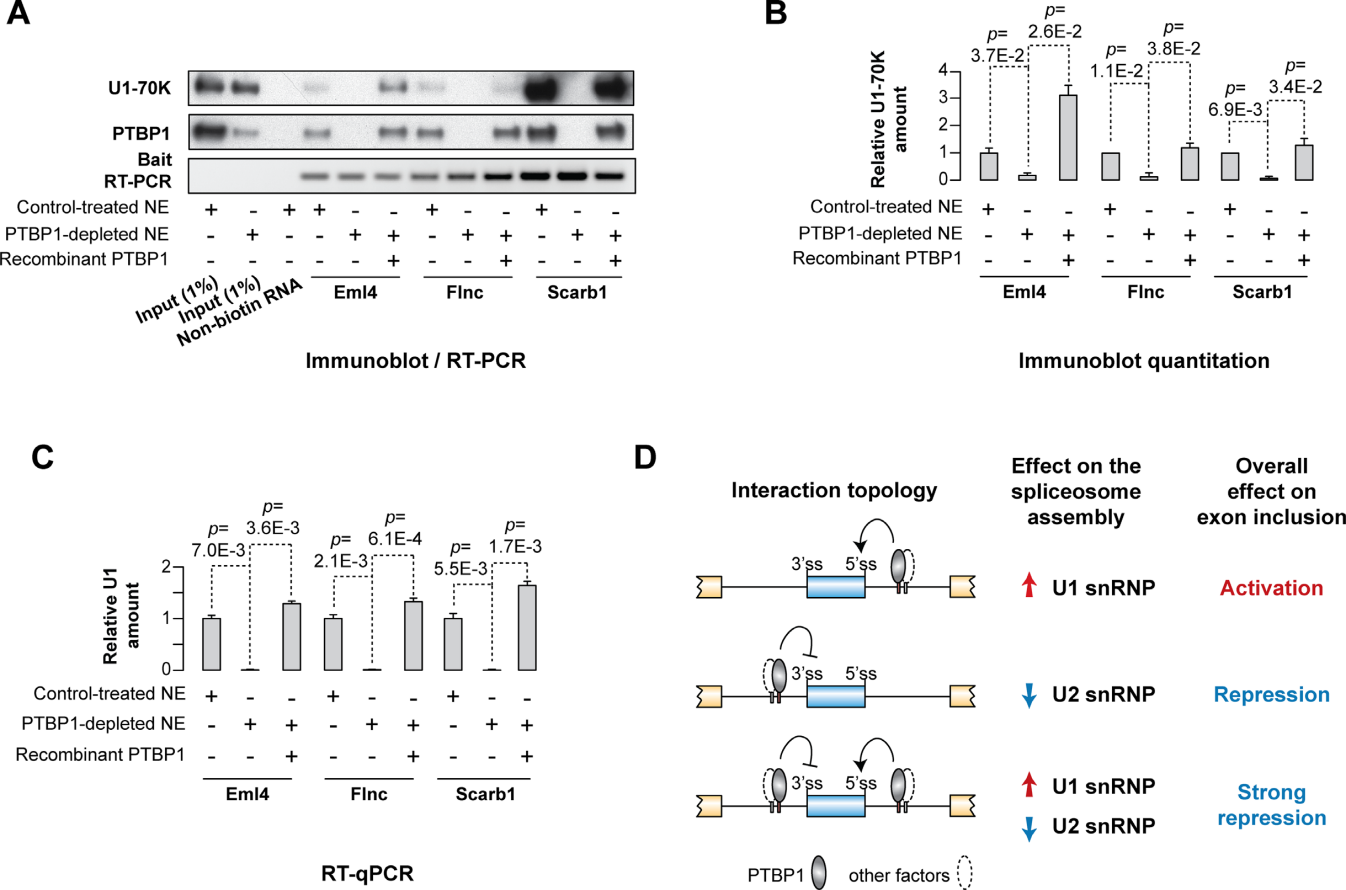
Augmented recruitment of U1 is a general mechanism for splicing activation by PTBP1. (**A**–**C**) Pull-down experiments were carried as outlined in Figure 4E–G using RNA baits containing 5’ss-adjacent sequences from the PTBP1-stimulated cassette exons from the *Eml4, Flnc* and *Scarb1* genes. (A) Immunoblot analysis with U1-70K and PTBP1-specific antibodies, (B) immunoblot quantitation and (C) RT-qPCR analysis of the U1 recruitment efficiency suggest that all three probes form stable complexes with U1 only in the presence of bound PTBP1. Data in (B and C) are quantified from 3 independent experiments ±SD and compared by a two-tailed t-test assuming unequal variances. (**D**) A mechanistic model for the choice between PTBP1-mediated activation and repression of alternative exons.

We also repeated the pull-down experiment using biotinylated RNAs comprising the natural PTBP1-dependent cassette exons from the *Eml4, Flnc* and *Scarb1* genes in their natural intronic contexts (see Supplementary Figure S3). Similar to the wild-type Dtx2 and the recombinant miniDtx2(SrcN1)-G4A-derived baits above, U1 snRNP was efficiently recruited to all three of these RNAs in untreated nuclear extract (Figure 7A and B). The recruitment was impaired by PTBP1 immunodepletion and rescued by the addition of purified PTBP1 back to the immunodepleted extract (Figure 7A and B). All in all, these data argue for generality of PTBP1-stimulated U1 recruitment as a splicing activation mechanism.

## DISCUSSION

The choice between inclusion and skipping of alternative exons involves integration of positive and negative inputs from *trans*-acting protein factors interacting with splicing enhancer and silencer elements. Mounting evidence suggests that many individual RBPs can function as either activators or repressors of splicing depending on their pre-mRNA binding position. Yet, how such positional information is decoded at the molecular level remains an open question. Here we identify a mechanism allowing PTBP1, an important post-transcriptional regulator with extensively characterized splicing repressor activities, to function as a bona fide activator of alternative exons.

We show that PTBP1 promotes inclusion of a cassette exon by interacting with downstream intronic pyrimidine-rich elements and facilitating productive recruitment of U1 snRNA to a suboptimal 5’ss (Figures 2D and E, 4, 5 and Supplementary Figure S8). This appears to be a general mechanism since PTBP1 is essential for efficient interaction of U1 with at least four natural and one recombinant pre-mRNA regulated in this manner (Figures 4, 7A–C and Supplementary Figure S12). These results are consistent with earlier studies that proposed that PTBP1 might stimulate cassette exons and alternative 5’ss directly without describing the underlying molecular mechanisms (28,33). Combined with evidence for an indirect mechanism used by PTBP1 to activate some alternative exons (25), our data suggest that the molecular effects of PTBP1 on pre-mRNA splicing are more diverse than previously thought.

Ptbp1 recruitment to a downstream intronic position was previously shown to facilitate repression of the SRC cassette exon N1 by forming physical contacts with the stem-loop element SL4 in the U1 snRNA (58). In that case, PTBP1 altered the U1 snRNP interaction footprint on the N1 5’ss and interfered with the assembly of a functional spliceosome (58). Our experiments with SL4 mutants known to strengthen (M10) or weaken (Dm) association of U1 with PTBP1 on the SRC pre-mRNA suggest that a similar interaction mechanism is likely used in the case of PTBP1-activated exons (Figure 3). This argues that interaction of PTBP1 with U1 is not an inherently repressive event and other mechanisms must determine the choice between the two opposite functional outcomes.

Since PTBP1-activated exons have significantly weaker 5’ss than their PTBP1-repressed counterparts, both in human (28) and mouse (Supplementary Figure S1B), one may hypothesize that the choice between repression and activation is determined by the degree of base paring between U1 and this element. Consistent with this possibility, splicing effects of hnRNPs L, a PTBP1-related protein, are known to be modulated by the 5’ss strength (61). Moreover, hnRNPs L and hnRNP A1 have been previously shown to repress some exons by extending contacts between U1 and the 5’ss and thus effectively blocking subsequent spliceosome maturation steps (62). However, strengthening U1 interaction with the 5’ss in the Dtx2 e6 context consistently enhanced inclusion of this exon in the presence of PTBP1 rather than promoting its skipping (Figure 3B). Thus, possible ‘hyper-stabilization’ of the U1/pre-mRNA complex cannot explain the switch from activation to repression in the case of PTBP1.

That said, at least two lines of evidence argue that the 5’ss has to be relatively weak to enable PTBP1-mediated splicing activation. (1) Substitution of the natural 5’ss with its consensus version in Dtx2 e6, used as our main experimental model, leads to constitutive inclusion of this exon in mouse cells (Figure 2A and B). (2) Efficient recruitment of U1 to the consensus 5’ss *in vitro* does not require PTBP1, unlike the situation with the natural Dtx2 e6 5’ss (Figure 4H–J). However, more subtle improvements in the base pairing between U1 and the 5’ss do not abolish the regulation pointing at its relative robustness (Figures 3F and 6A and B).

Many PTBP1-repressed exons including the SRC N1 contain both upstream and downstream PTBP1 binding elements (34,35,60). When we recapitulated this arrangement by adding the SRC-derived upstream PTBP1-interacting sequence to a Dtx2 e6-containing minigene containing the natural downstream Py elements, the behavior of e6 changed from PTBP1 activation to repression (Figure 6A–B and Supplementary Figure S11B and C). A similar effect has been observed earlier for another cassette exon naturally stimulated by PTBP1 (28).

Our data shed some light on molecular details underlying this functional switch. We show that upstream PTBP1 interaction sequences do not compromise PTBP1-facilitated recruitment of U1 but instead hinder U2 binding (Figure 6C and D and Supplementary Figure S11D). This result is generally consistent with previously published functional data (23) and biochemical analyses of a recombinant exon flanked by PTBP1-binding sequences (40). Of note, the e6 inclusion efficiency in the upSrc-Py123mut and the up2Src-Py123mut transcripts containing only upstream PTBP1-binding elements is substantially higher compared to their upSrc and up2Src counterparts where such elements are located both upstream and downstream of the regulated exon (Figure 6C and D, Supplementary Figures S10 and S11D). This is somewhat surprising given that U1 interacts with upSrc-Py123mut and up2Src-Py123mut significantly worse than with upSrc and up2Src (Figure 6C and D and Supplementary Figure S11D).

A model emerging from these results is summarized in Figure 7D. In the absence of other regulatory interactions, PTBP1 recruited downstream of an alternative exon stimulates its inclusion by stabilizing productive U1 docking to the 5’ss. Exclusive recruitment of PTBP1 upstream of the exon can promote its skipping, at least in part by antagonizing U2 binding to the upstream branch point. Finally, binding of PTBP1 on both sides of a regulated exon is capable of inducing a strong repressive effect that might involve assembly of an aberrant exon definition complex.

Although additional work will obviously be required to explore the latter possibility, PTBP1 binding to intronic sequences flanking an alternative exon has been recently shown to interfere with normal exon definition by allowing recruitment of the U1 but not the U2 snRNP (40). The overall composition of proteins interacting with the exon repressed in this manner differs markedly from that of the normally defined exon (40). Therefore, the non-additive outcome of combining the upstream and the downstream PTBP1 sequences in the upSrc and up2Src minigenes (Figure 6, Supplementary Figures S10 and S11) might be mediated by sequestering e6 in a non-productive ribonucleoprotein complex that effectively competes with proper definition of this exon. This will be an interesting line of research to pursue in the future, especially given the increasing appreciation of ‘trapped’ states in the spliceosome assembly and the roles they play in splicing regulation (40,62–64).

In addition to PTBP1, several other RBPs have been shown to either activate or repress splicing by forming contacts with U1 snRNP in the vicinity of their binding sites (62,65–69). Our study extends this line of research by showing that the functional consequence of such contacts can be modulated to a remarkable degree by additional interactions of the same RBP with its pre-mRNA target. This argues that the information encoded by individual RBP binding events is fundamentally insufficient for instructing the ultimate splicing decisions and the exactly same RNA element may function as an enhancer or silencer depending on a wider sequence context. If so, considering possible crosstalk between interaction sites in a systematic manner should substantially enhance the power of future knowledge-based models aiming to predict splicing changes that occur in response to disease-associated mutations or as a result of evolution.

## Supplementary Material

Supplementary Data
