# Supplementary material for: A mechanism underlying position-specific regulation of alternative splicing
Source: Nucleic Acids Res. 2017 Oct 9;45(21):12455–68. doi: 10.1093/nar/gkx901 (PMC5716086; doi:10.1093/nar/gkx901)
Supplement: Supplementary Data [file gkx901_supp.zip › nar-02048-a-2017-File009.pdf]

Supplementary Material

A mechanism underlying position-specific regulation of alternative splicing

Fursham M. Hamid and Eugene V. Makeyev

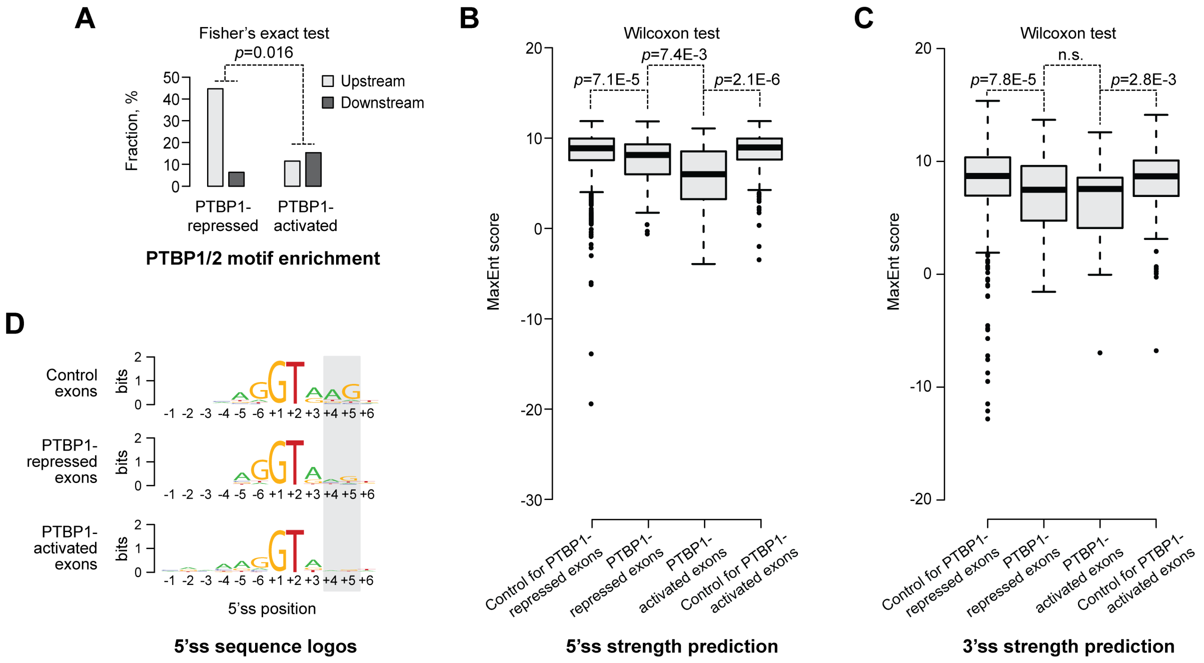

Supplementary Figure S1. PTBP1-activated exons are often followed by intronic PTBP1 sequence motifs and have relatively weak 5'ss. (**A**) Compared to the PTBP1-repressed exons, PTBP1 interaction motifs are depleted upstream and enriched downstream of the activated exons. **(B)** MaxEnt predictions showing that the 5'ss of PTBP1-activated exons are significantly weaker as compared to the PTBP1-repressed and non-regulated exons. (**C**) MaxEnt scores for 3'ss do not differ significantly between the activated and repressed exons although being relatively low in comparison with the corresponding non-regulated controls (**D**) WebLogo analysis showing that the 5'ss of the PTBP1-activated exons diverges from the MAG|GTRAGT consensus to a larger extent than the 5'ss of their PTBP1-repressed and non-regulated counterparts. Especially apparent is the lack of the conserved AG dinucleotide at the +4+5 positions (shaded area).

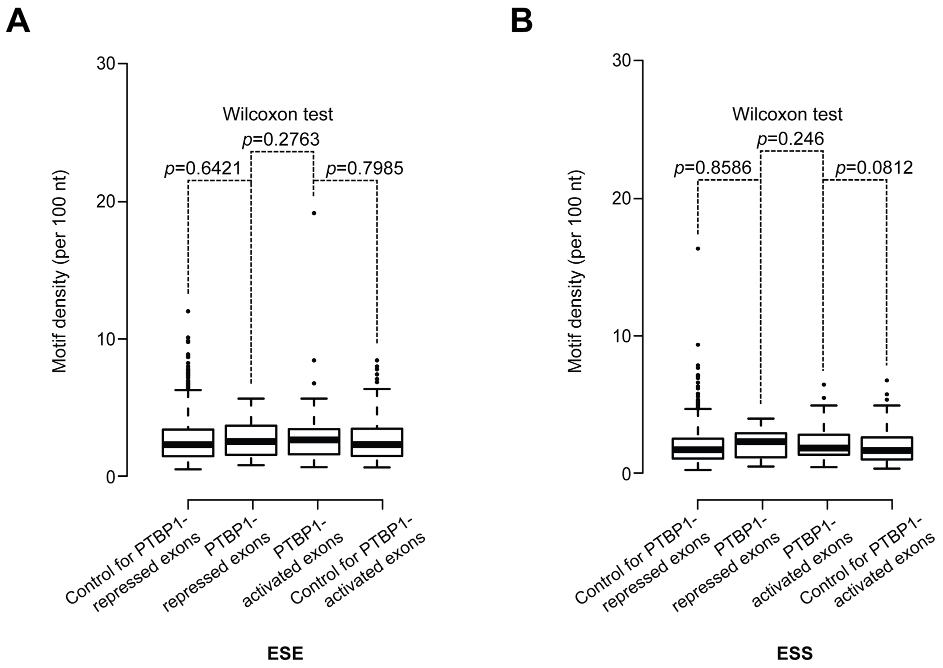

Supplementary Figure S2. Bioinformatics analyses showing that densities of (**A**) exonic splicing enhancer (ESE) and (**B**) exonic splicing silencer (ESS) motifs do not differ among PTBP1-activated and PTBP1-repressed and non-regulated exons.

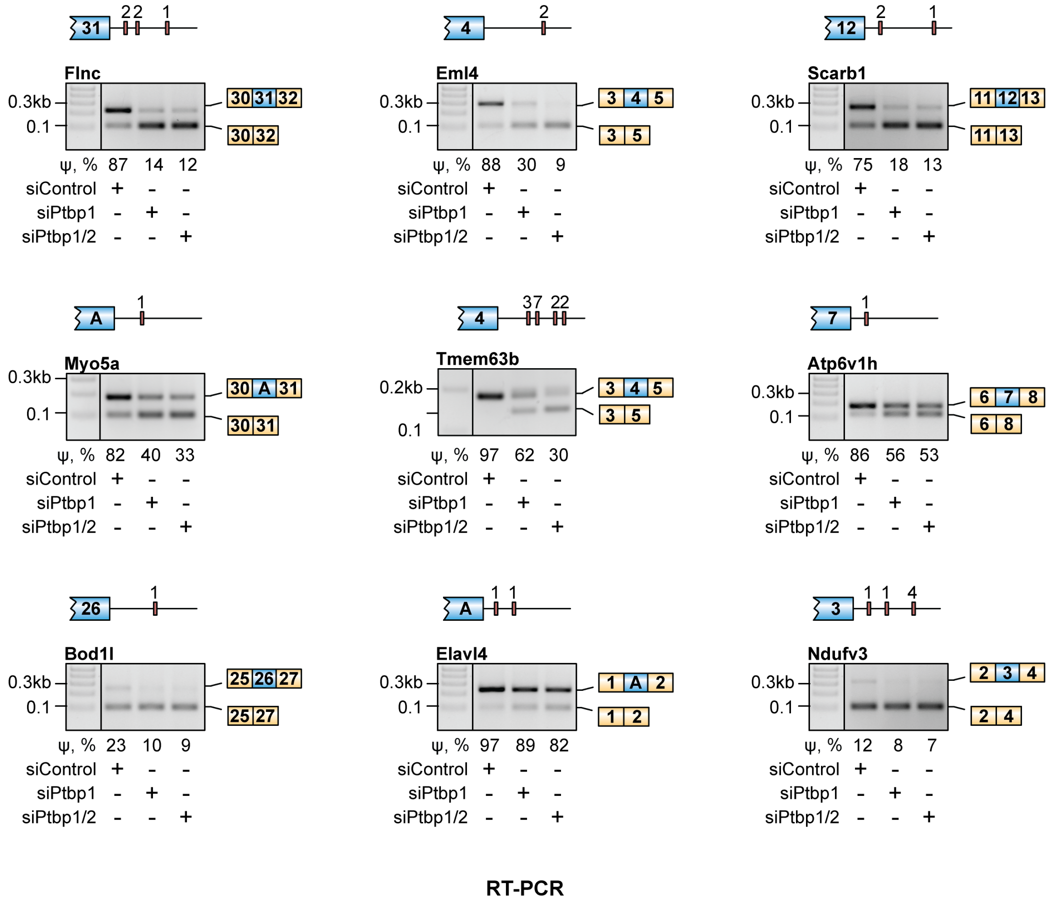

Supplementary Figure S3. Experimental validation of PTBP1-activated exons. Selected examples of bioinformatically identified PTBP1-activated cassette exons were validated by either regular or multiplex RT-PCR analyses of CAD cells treated with siControl, siPtbp1 or siPtbp1/2. The following primer mixtures were used: Flnc, Flnc_F1 and Flnc_R2; Eml4, Eml4_F1 and Eml4_R2; Scarb1, Scarb1_F1 and Scarb1_R2; Myo5a, Myo5a_F1 and Myo5a_R2; Tmem63b, Tmem63b_F1 and Tmem63b_R2; Atp6v1h, Atp6v1h_F1 and Atp6v1h_R2; Bod1l, Bod1l_F1 and Bod1l_R2; Elavl4, Elavl4_F1, Elavl4_F2 and Elavl4_R1; Ndufv3, Ndufv3_F1, Ndufv3_F2 and Ndufv3_R1. Diagrams on the top of each panel indicate positions of putative PTBP1 recognition motifs (YTCTCY, YCTCTY, YTCTTY, YTTCTY) within 150 nt intronic windows following the regulated exons. Regulated exon-specific percent splice in (ψ) values are shown at the bottom.

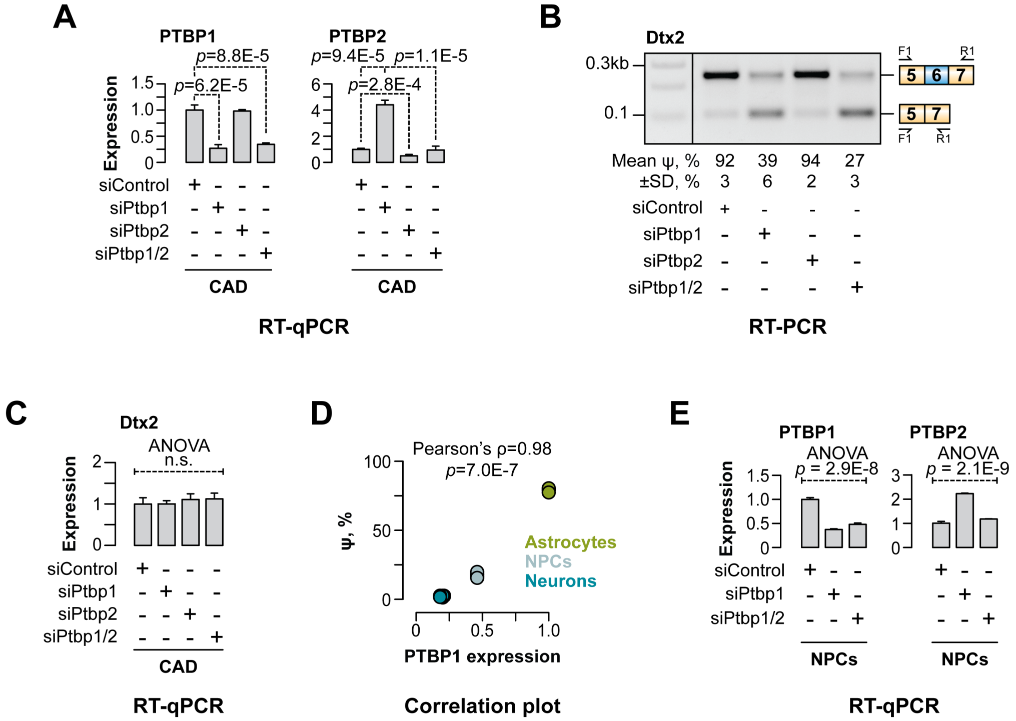

Supplementary Figure S4. Relationship between PTBP1, PTBP2 and Dtx2 expression levels. (**A**) CAD cells were treated with siControl, siPtbp1, siPtbp2 or both siPtbp1 and siPtbp2 (siPtbp1/2) and analyzed by RT-qPCR with PTBP1- and PTBP2-specific primers (Table S4). Note that siPtbp1 down-regulates its target ~4-fold compared to the siControl samples. When added alone, siPtbp1 additionally leads to a ~4.5-fold increase in the PTBP2 levels, as reported previously (1-4). This effect is alleviated in the siPtbp1/2 samples leading to relatively low expression of both PTBP1 and PTBP2. (**B**) CAD cells treated in (A) were analyzed by RT-PCR for Dtx2e6 splicing pattern. Compared to siControl, siPtbp1 promotes e6 skipping and this effect becomes even more apparent following down-regulation of both PTBP1 and PTBP2 (siPtbp1/2). On the other hand, siPtbp2 does not alter the e6 inclusion in the absence of siPtbp1. This is expected given that PTBP2 is expressed at extremely low levels in the presence of PTBP1. (**C**) RT-qPCR with EMO4892 and EMO4893 primers followed by one-way ANOVA analysis shows that the above siRNAs do not change the overall expression levels of the Dtx2 mRNA in CAD cells. (**D**) A scatter plot showing a strong positive correlation between PTBP1 expression levels and the efficiency of e6 inclusion into Dtx2 mRNA (ψ) in the neural cell lineage including astrocytes, neuronal progenitor cells and neurons. (**E**) RT-qPCR validation of PTBP1 and PTBP2 knockdown efficiencies in NPCs treated with the indicated siRNAs. Data in (A, B and D) are averaged from three independent experiments ±SD.

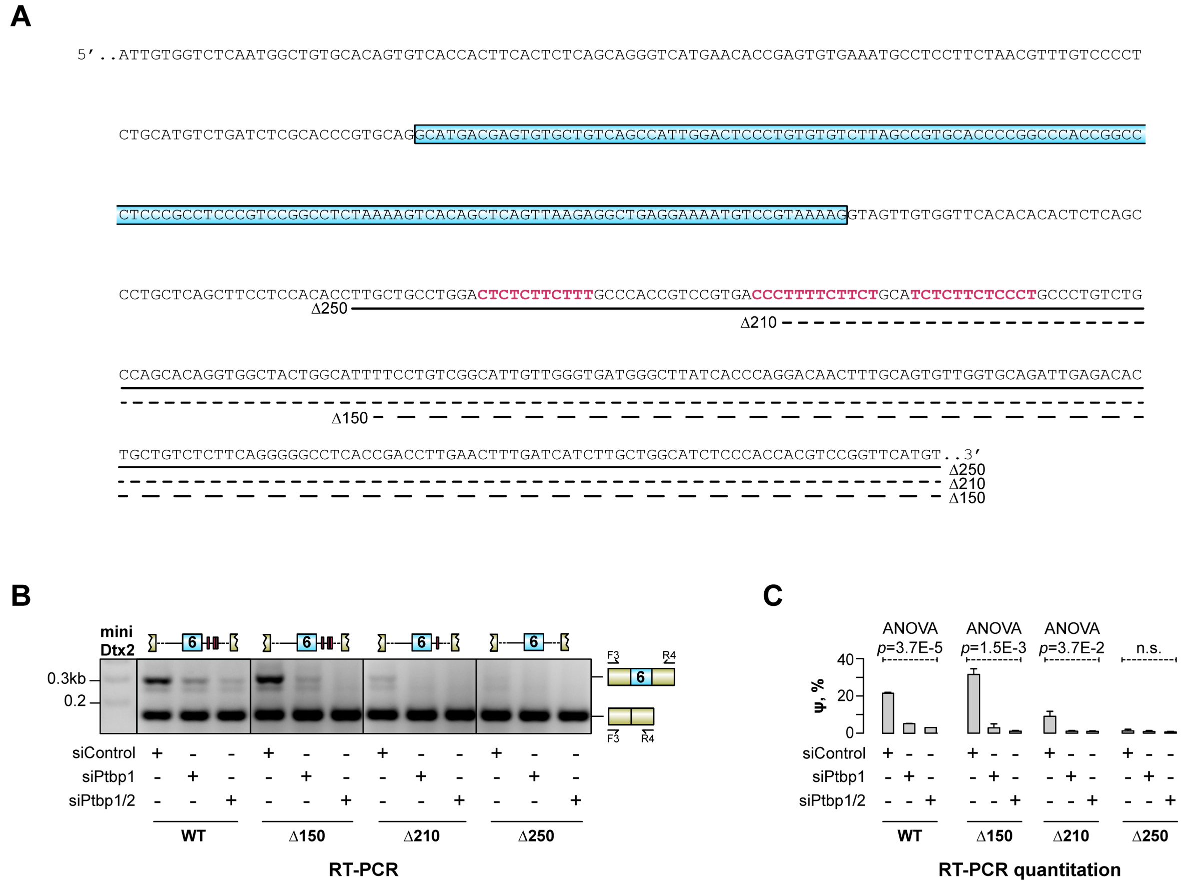

Supplementary Figure S5. Inclusion of e6 requires downstream intronic sequences. (**A**) Dtx2 e6 sequence (blue box) in its natural intronic context. Deletions generated in this study are underlined and pyrimidine-rich elements are shown in red. (**B**) CAD cells pretreated with the indicated siRNAs were transfected with either the wild-type (WT) miniDtx2 construct or its derivatives lacking the downstream intronic sequences underlined in (A). Note that e6 remains fully responsive to PTBP1 in the minigene lacking a 150 nt-long intronic segment and partially responsive in the Δ210 version. Deletion of 250 nt leads to constitutive skipping of e6 thus suggesting that the 50-150 nt sequence window downstream of e6 contains splicing PTBP1/2-dependent splicing enhancers. (**C**) The experiment in (B) was repeated in total 3 times and the e6-specific ψ values were plotted as mean±SD and analyzed by one-way ANOVA.

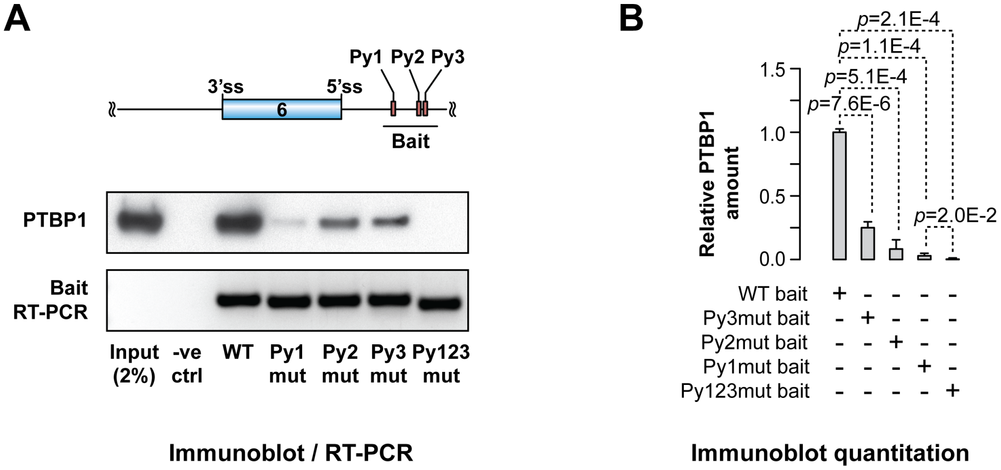

Supplementary Figure S6. All three Py elements are required for optimal binding of PTBP1 to the Dtx2 pre-mRNA. (**A**) Biotinylated RNA probes ("baits") corresponding to the Dtx2 intronic region indicated on the top and containing either the wild-type (WT) or mutated Py sequences were incubated with HeLa nuclear extract, and the efficiency of their interaction with PTBP1 protein was analyzed by immunoblotting (bottom). Unlabeled WT Dtx2 RNA was used as a negative control (-ve ctrl) and lane loading was estimated by bait-specific RT-PCR. (**B**) Quantitation of the data in (A) averaged from three independent experiments ±SD and compared by a two-tailed t-test assuming unequal variances. Note that inactivation of individual Py elements leads to a pronounced decrease in the overall PTBP1 binding efficiency with the relative effects of the three mutations decreasing in the Py1mut>Py2mut>Py3mut order. No PTBP1 binding is detected when all three Py's are mutated simultaneously (Py123mut).

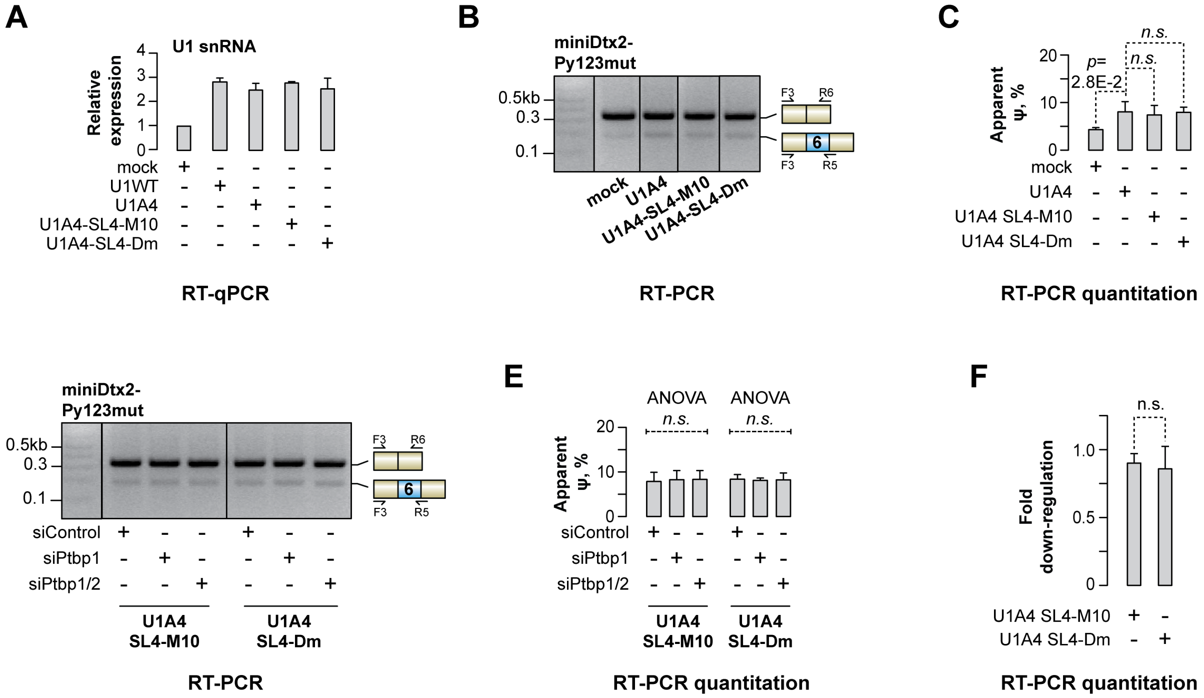

Supplementary Figure S7. Controls for U1 suppressor experiments. (**A**) RT-qPCR analysis of the U1 expression levels in CAD cells co-transfected with the miniDtx2-WT minigene and an empty vector (mock) or corresponding expression constructs encoding a wild-type U1 (U1WT) or its derivatives (U1A4, U1A4-SL4-M10 or U1A4-SL4-Dm) (see Fig. 3C-G). Note that all four U1-encoding constructs lead to a comparable increase in the total cellular levels of this snRNA. (**B**) CAD cells were co-transfected with miniDtx2-Py123mut and either an expression construct encoding a modified U1 snRNA (U1A4, U1A4-SL4-M10 or U1A4-SL4-Dm) or an empty vector (mock) and analyzed by a multiplex RT-PCR with F3/R5/R6 primers affording more sensitive detection of the minigene-derived e6-containing transcripts that the regular RT-PCR used in Fig. 2D. Note that U1A4 promotes e6 inclusion compared to the mock but, unlike the situation with the miniDtx2-WT (Fig. 3D), the U1A4-SL4-M10 and the U1A4-SL4-Dm samples are indistinguishable from U1A4. (**C**) The experiment in (B) was repeated in total 3 times and apparent ψ values for the minigene-encoded e6 were plotted as mean±SD and compared using a two-tailed t-test assuming unequal variances. (**D**) CAD cells pretreated with siControl, siPtbp1 or siPtbp1/2 were co-transfected with miniDtx2-Py123mut and either U1A4-SL4-M10 or U1A4-SL4-Dm and analyzed by the multiplex RT-PCR approach introduced in (B). (**E**) Repeating the experiment in (D) 3 times and comparing apparent ψ values for the minigene-encoded e6 by one-way ANOVA confirms that miniDtx2-Py123mut remains non-responsive to PTBP1/2 in the presence of U1A4-SL4-M10 or U1A4-SL4-Dm. (**F**) A similar conclusion can be drawn from the comparison of the siPtbp1/2 fold down-regulation effects on the e6 inclusion into the miniDtx2-Py123mut transcripts carried out to match a similar analysis in Fig. 3G.

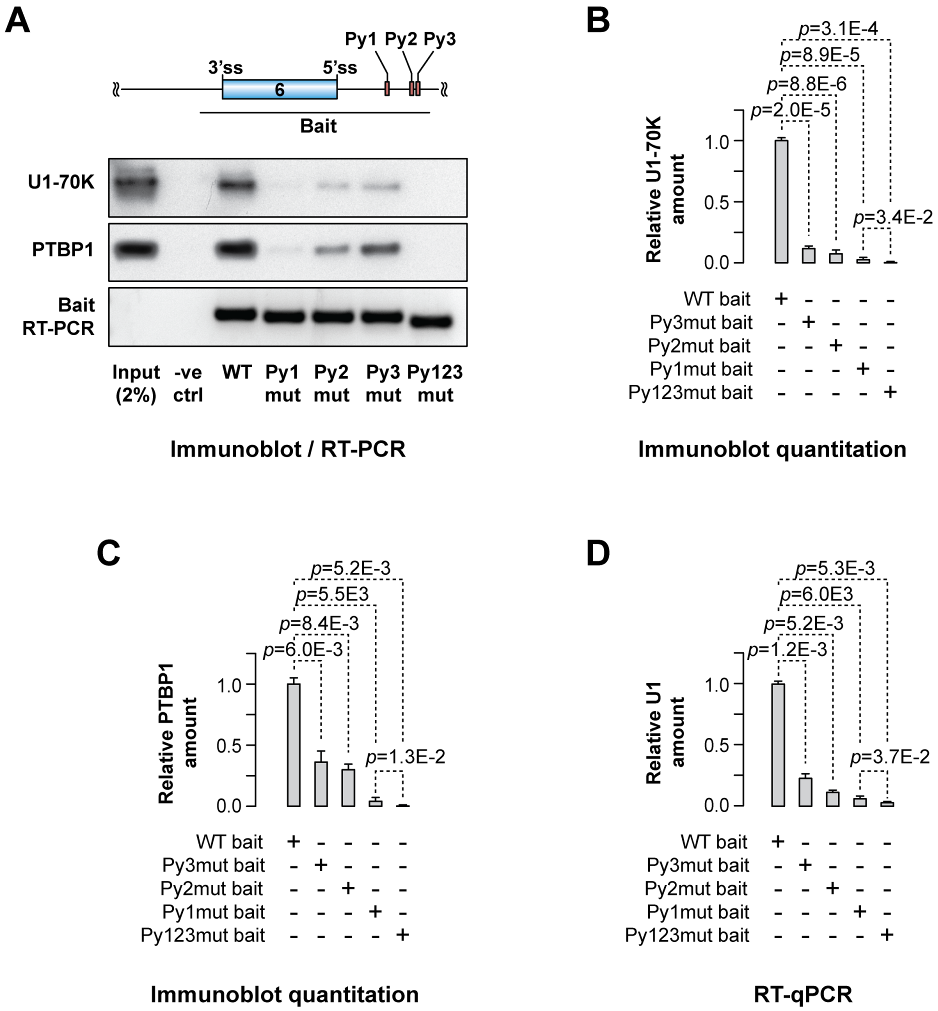

Supplementary Figure S8. Stimulation of U1 snRNP recruitment by PTBP1 depends on the Py elements. (**A**) Biotinylated RNA baits containing e6 in its immediate intronic context with either natural (WT) or mutant Py sequences (top) were incubated with HeLa nuclear extract and their interaction with U1 snRNP and PTBP1 was analyzed by immunoblotting using indicated antibodies (bottom). Unlabeled WT Dtx2 RNA was used as a negative control (-ve ctrl) and lane loading was controlled by bait-specific RT-PCR. Py mutations lead to a progressive loss of PTBP1 binding with the effect diminishing in the following order: Py123mut>Py1mut>Py2mut>Py3mut, similar to the data in Fig. S6. Consistent with the dependence of U1 snRNP recruitment on PTBP1, U1-70K interaction with the baits follows a similar trend. (**B-C**) Quantitation of (B) U1-70K and (C) PTBP1 recruitment efficiencies in (A). (**D**) U1 snRNP recruitment in the experiment described in (A) was additionally analyzed by RT-qPCR with U1 snRNA-specific primers. Data in (B-D) were averaged from three independent experiments ±SD and analyzed by a two-tailed t-test assuming unequal variances.

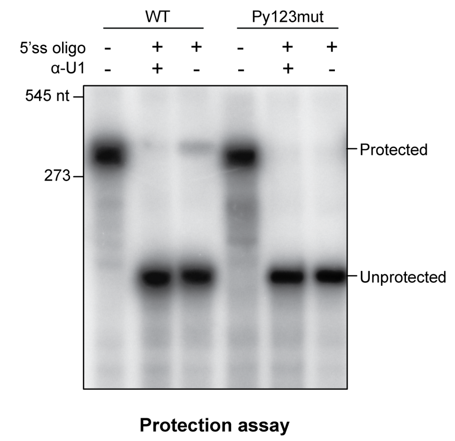

Supplementary Figure S9. PTBP1 recruits U1 snRNP to the 5'ss. ^{32}P-labeled WT or Py123mut probes designed as outlined in Fig. 4 were incubated with the indicated oligonucleotides, HeLa nuclear extract and RNase H and the reaction products were analyzed by polyacrylamide gel electrophoresis under denaturing conditions. Note that following RNase H cleavage only the 5'-terminal "unprotected" RNA fragment is detected possibly because it is capped and therefore more stable than the 3'-terminal product.

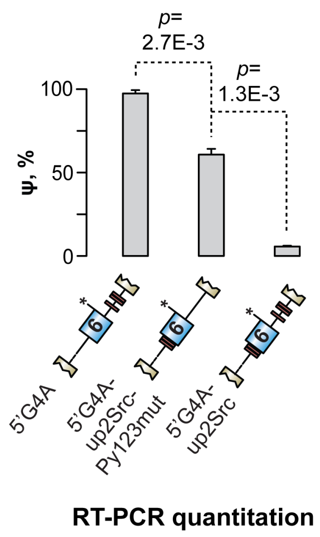

Supplementary Figure S10. Quantitation of the e6 inclusion efficiency for miniDtx2-5'G4A, miniDtx2-5'G4A-upSrc-Py123mut and miniDtx2-5'G4A-upSrc minigene transcripts. Data are averaged from 3 independent experiments ±SD and compared by a two-tailed t-test assuming unequal variances. See Fig. 6A for further details.

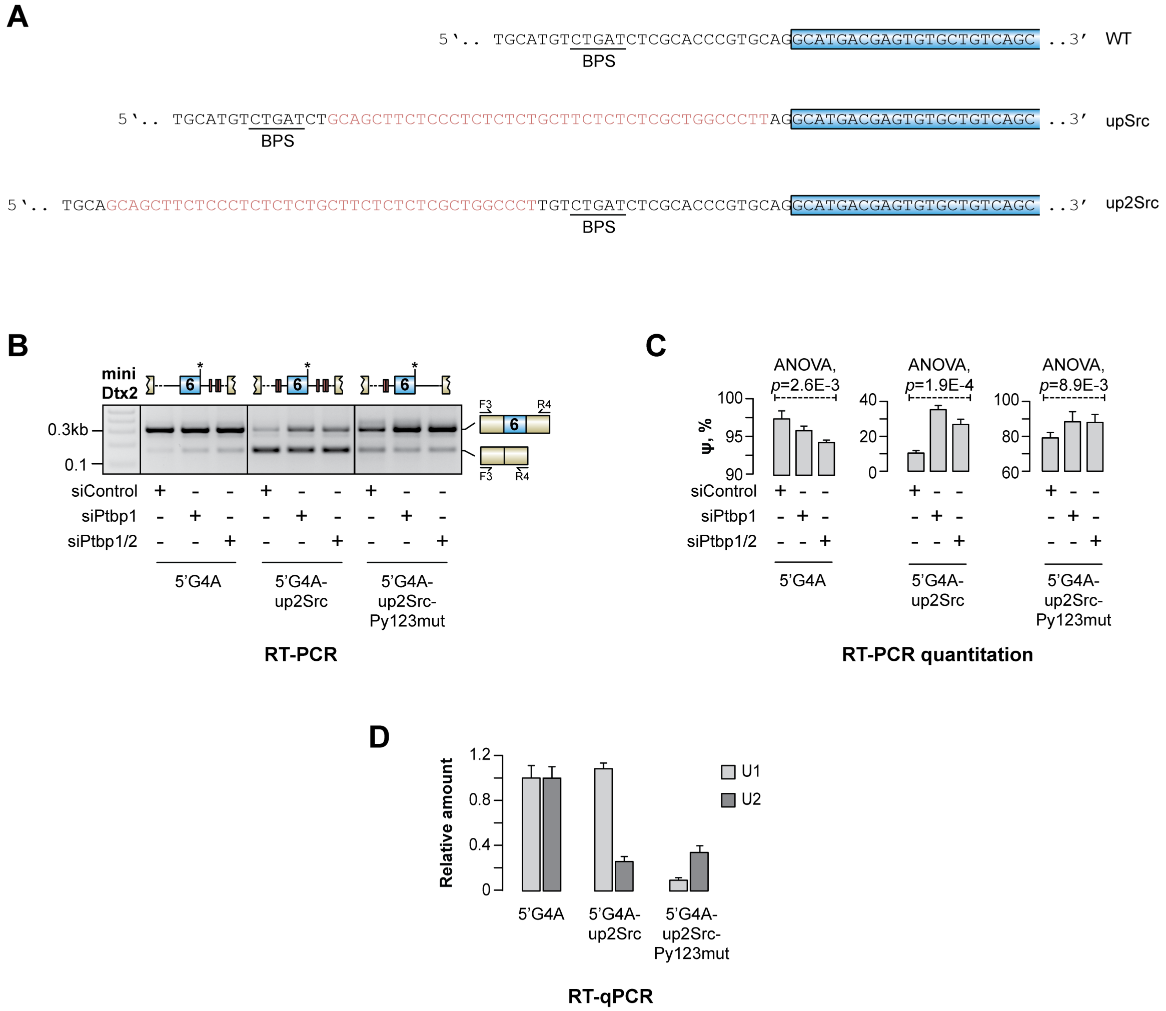

Supplementary Figure S11. PTBP1 binding both upstream and downstream of Dtx2 e6 leads to a strong repression effect. (**A**) The wild-type intronic sequence preceding Dtx2 e6 (WT) and its recombinant derivatives containing PTBP1-intercacting element from the human *SRC* gene (red sequence) either substituting the Dtx2-specific 3'ss region (upSrc) or inserted in front of the predicted branch point sequence (BPS; underlined) (up2Src). (**B**) CAD cells treated with siControl, siPtbp1 and siPtbp1/2 were transfected with 5'G4A versions of Dtx2 minigenes containing wild-type (mini-Dtx2-5'G4A) or mutated intronic sequences (mini-Dtx2-5'G4A-up2Src and miniDtx2-5'G4A-up2Src-Py123mut). Note that the addition of the PTBP1-interaction sequence in front of the BPS converts e6 into a PTBP1-repressed exon. The repression is noticeably stronger when PTBP1 can bind both upstream and downstream of e6 (mini-Dtx2-5'G4A-up2Src) compared to the situation when only upstream binding is possible (miniDtx2-5'G4A-up2Src-Py123mut). (**C**) Quantitation of the data in (B) averaged from three independent experiments ±SD and analyzed by one-way ANOVA. (**D**) Biotinylated-RNA baits corresponding to the 5'G4A-up2Src minigene series were assayed for their ability to interact with the U1 and the U2 snRNPs as described in Fig. 6C. Similar to the upSrc data (Fig. 6C), the up2Src modification reduces the U2 recruitment efficiency while having no effect on U1 binding. Optimal U1 binding depends on the downstream Py elements, as expected.

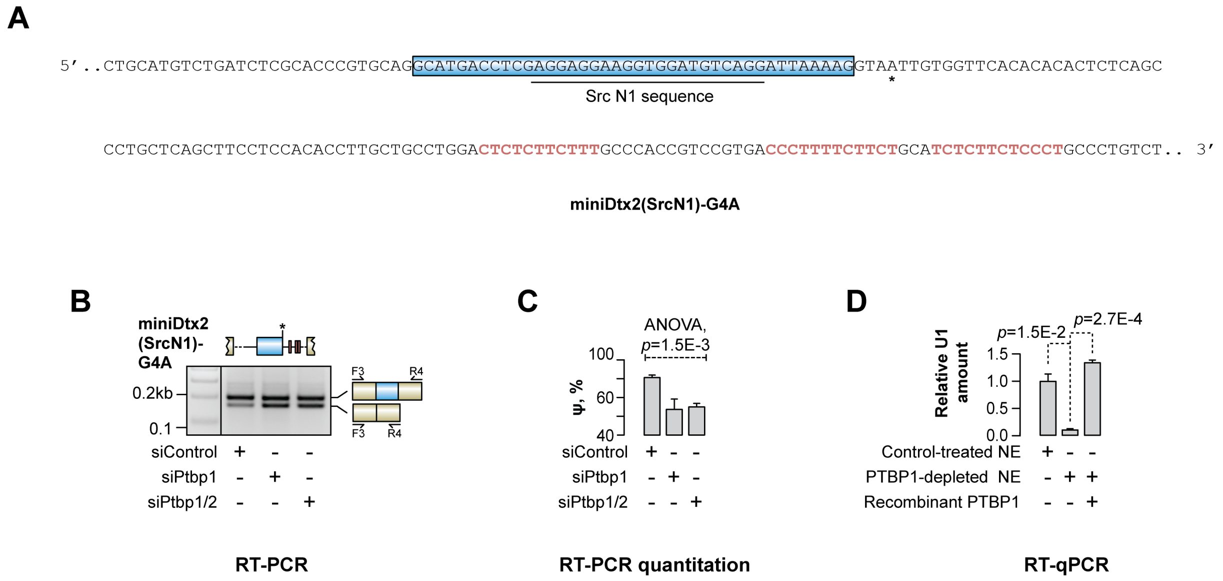

Supplementary Figure S12. PTBP1 promotes inclusion of a recombinant exon containing SRC N1 sequence in Dtx2-specific context. (**A**) A diagram of the miniDtx2(SrcN1)-G4A minigene derived from miniDtx2-5'G4A by replacing most of Dtx2 e6 with a SRC N1 sequence (underlined). The Dtx2-specific Py elements are shown in red. (**B**) CAD cells pretreated with siControl, siPtbp1 or siPtbp1/2 were transfected with miniDtx2(SrcN1)-G4A and the minigene-specific splicing pattern was analyzed by RT-PCR. Note that PTBP1 promotes inclusion of the recombinant exon. (**C**) Quantitation of the exon inclusion efficiency in (B) averaged from three independent experiments ±SD and analyzed by one-way ANOVA. (**D**) Pull-down/RT-qPCR assay showing that a biotinylated RNA bait corresponding to miniDtx2(SrcN1)-G4A and designed otherwise as in Fig. 4 recruits U1 in a PTBP1-dependent manner. Data are averaged from 3 amplification experiments ±SD and compared by a two-tailed t-test assuming unequal variances.

Supplementary References

1. Makeyev, E.V., Zhang, J., Carrasco, M.A. and Maniatis, T. (2007) The MicroRNA miR-124 promotes neuronal differentiation by triggering brain-specific alternative pre-mRNA splicing. *Mol Cell*, **27**, 435-448.
2. Boutz, P.L., Stoilov, P., Li, Q., Lin, C.H., Chawla, G., Ostrow, K., Shiue, L., Ares, M., Jr. and Black, D.L. (2007) A post-transcriptional regulatory switch in polypyrimidine tract-binding proteins reprograms alternative splicing in developing neurons. *Genes Dev*, **21**, 1636-1652.
3. Spellman, R., Llorian, M. and Smith, C.W. (2007) Crossregulation and functional redundancy between the splicing regulator PTB and its paralogs nPTB and ROD1. *Mol Cell*, **27**, 420-434.
4. Yap, K., Lim, Z.Q., Khandelia, P., Friedman, B. and Makeyev, E.V. (2012) Coordinated regulation of neuronal mRNA steady-state levels through developmentally controlled intron retention. *Genes Dev*, **26**, 1209-1223.

Supplementary Tables

Table S1. Cassette exons consistently repressed by PTBP1 in CAD cells.

Table S2. Cassette exons consistently activated by PTBP1 in CAD cells.

Table S3. Plasmids generated in this study.

Table S4. Primers used in this study.
